# Activation of GCN2 by the ribosomal P-stalk

**DOI:** 10.1073/pnas.1813352116

**Published:** 2019-02-25

**Authors:** Alison J. Inglis, Glenn R. Masson, Sichen Shao, Olga Perisic, Stephen H. McLaughlin, Ramanujan S. Hegde, Roger L. Williams

**Affiliations:** ^a^Medical Research Council Laboratory of Molecular Biology, Cambridge CB2 0QH, United Kingdom

**Keywords:** GCN2, P-stalk, HDX-MS, uL10/P1/P2, ribosome stalling

## Abstract

Cells dynamically adjust their protein translation profile to maintain homeostasis in changing environments. During nutrient stress, the kinase general control nonderepressible 2 (GCN2) phosphorylates translation initiation factor eIF2α, initiating the integrated stress response (ISR). To examine the mechanism of GCN2 activation, we have reconstituted this process in vitro, using purified components. We find that recombinant human GCN2 is potently stimulated by ribosomes and, to a lesser extent, by tRNA. Hydrogen/deuterium exchange–mass spectrometry (HDX-MS) mapped GCN2–ribosome interactions to domain II of the uL10 subunit of the ribosomal P-stalk. Using recombinant, purified P-stalk, we showed that this domain of uL10 is the principal component of binding to GCN2; however, the conserved 14-residue C-terminal tails (CTTs) in the P1 and P2 P-stalk proteins are also essential for GCN2 activation. The HisRS-like and kinase domains of GCN2 show conformational changes upon binding recombinant P-stalk complex. Given that the ribosomal P-stalk stimulates the GTPase activity of elongation factors during translation, we propose that the P-stalk could link GCN2 activation to translational stress, leading to initiation of ISR.

## Significance

General control nonderepressible 2 (GCN2) phosphorylates eIF2α, regulating translation in response to nutritional stress. Here, we show that although tRNA stimulates purified, recombinant human GCN2 in vitro, mammalian ribosomes are even more potent GCN2 activators. Hydrogen/deuterium exchange–mass spectrometry (HDX-MS) showed GCN2 interacting with domain II of the uL10 P-stalk protein. The P-stalk is a uL10/P1_2_/P2_2_ pentameric complex that is part of the ribosomal GTPase-associated center. Recombinant human P-stalk greatly stimulates GCN2. Both domain II of uL10 and the C-terminal tails of P1 and P2 are necessary for maximal GCN2 activation. On actively translating ribosomes, the C-terminal tails of P1 and P2 are sequestered by elongation factors, suggesting P-stalk availability could link translational stress to GCN2 activation.

A dynamic balance of anabolic and catabolic cellular pathways interlace to efficiently use available resources. The integrated stress response (ISR) enables cells to recognize a variety of imbalances in key cellular resources and respond by activating protein kinases that modify the cell’s translational and transcriptional programs. The ISR is widely conserved across eukaryotes and responds to numerous stressors including amino acid insufficiency, protein misfolding, high salinity, UV light, and glucose starvation (1). General control nonderepressible 2 (GCN2) is one of four related kinases that respond to these cellular stresses by phosphorylating the translation initiation factor eIF2α (2). All four eIF2α kinases have a conserved catalytic domain, but each contains additional regulatory domains enabling specificity of activation. The most commonly studied stress leading to GCN2 activation is amino acid starvation. GCN2 is implicated in a number of biological processes in both health and disease, including the development of neurological disorders (3), the onset of pulmonary veno-occlusive disease (4), and the growth of Ras-transformed tumors under conditions of nutrient deprivation (5).

The eIF2 (αβγ) heterotrimer, together with GTP and the initiator methionyl-tRNA^Met^, forms a ternary complex that delivers the initiator tRNA to the small ribosomal subunit to form the 43S preinitiation complex (6). Translation initiation causes the release of GDP-bound eIF2, which is then recycled to a GTP-bound state by the guanine exchange factor (GEF) eIF2B. Under stress conditions, eIF2α phosphorylation converts the translation initiation factor from a substrate to a competitive inhibitor of eIF2B, thereby decreasing global translation and conserving nutrients and energy. A select number of mRNAs are able to bypass this translational block, such as the mRNA for the transcription factor ATF4 (GCN4 in yeast) that up-regulates transcription of various genes involved in the stress response (7–9).

How GCN2 recognizes and responds to amino acid starvation has been a subject of investigation for over three decades. In prokaryotes, the stringent response is a mechanism whereby amino acid availability is signaled via alarmone nucleotides (p)ppGpp (10). Under amino acid starvation, cognate deacylated tRNA binds in the ribosome acceptor site (A site), recruits RelA to the ribosome, and stimulates RelA-mediated (p)ppGpp synthesis. This ribosome-mediated nutrient-sensing mechanism led to early efforts to examine whether GCN2 could be analogously regulated. Yeast GCN2 comigrates with ribosomes in sucrose gradients and a C-terminal domain (CTD) binds directly to the 60S ribosome subunit (11). Mutations in this domain eliminate ribosome binding and activation of yeast GCN2 in starved cells (12). The presence of a domain homologous to His-tRNA synthetase (HisRS-like) adjacent to the kinase domain of GCN2, together with the observation that the HisRS-like domain binds a broad range of deacylated tRNAs in preference to charged tRNAs, led to the proposition that GCN2 is regulated by binding deacylated tRNA, in a similar manner to RelA (13, 14). Mutation of two residues in the HisRS-like domain that are analogous to residues important for tRNA binding in tRNA synthetases generated the GCN2 mutant known as *m2* (13). This mutant greatly decreases binding to deacylated tRNA, decreases activity in vitro, and completely abolishes GCN2 activation in cells. Studies using this *m2* mutant have helped demonstrate the importance of uncharged tRNA (e.g., refs. 15–17). In addition to the *m2* mutant, a number of other mutations in the HisRS-like domain either constitutively activate GCN2 in yeast or impair tRNA binding and abolish activation in cells (17, 18). However, direct activation of wild-type yeast GCN2 in vitro by deacylated tRNA could not be demonstrated (15). More recent work with mammalian GCN2 did show a modest activation of GCN2 with tRNA in vitro (16, 19). For high-level nutritional sensing in yeast, GCN2 must associate with the GCN1/GCN20 regulatory complex, with GCN1 and GCN2 directly interacting with ribosomes (20, 21). GCN1 and GCN20 each have a domain that is related to regions of EF3, a fungal-specific protein involved in removing the uncharged tRNA from the ribosomal exit site (E site) during translation. This led to a model in which GCN1 and GCN20 would mimic the function of EF3; however, instead of removing an uncharged tRNA from the E site, it was proposed that GCN1 would remove an uncharged tRNA from the A site and transfer it to the HisRS-like domain of GCN2 (20, 22). More recent studies have identified additional direct activators of GCN2 that, similarly to tRNA, have their effects significantly ablated by the *m2* HisRS-like domain mutation. These include free cytosolic yeast P1 and P2 proteins of the ribosomal P-stalk (16) and Sindbis virus and HIV-1 genomic RNA (19, 23).

While GCN2 can be activated in cells, a wide range of observations suggest that the enzyme is maintained in an inactive state in the absence of stimulation (15, 17). Yeast GCN2 forms a constitutive dimer even in the absence of activation, principally through the CTD (24, 25). However, it has been proposed that the nature of the dimer is important for regulating the enzyme, with the active GCN2 dimer likely to have a parallel arrangement, and an inactive dimer having an antiparallel arrangement, as was observed in the crystal structure of the isolated GCN2 kinase domain (26–28). Binding to deacylated tRNA molecules in times of amino acid starvation has been suggested to cause a conformational rearrangement that alters multiple interdomain interactions resulting in activation and autophosphorylation of the GCN2 kinase domain (17, 29, 30).

The initial observation that yeast GCN2 associates with ribosomes and, in particular, with active polysomes (11), raised the possibility of an analogy with the action of RelA on prokaryotic ribosomes; however, the function of the ribosomal association has remained unclear. This lack of clarity was further confounded by a more recent report that, unlike yeast, mouse GCN2 does not form a stable complex that copurifies with ribosomes (24). New insight into a possible functional link between GCN2 and ribosomes came from a recent analysis of mice lacking both a specific neuronal tRNA (tRNA^Arg^_UCU_) and the putative ribosome recycling factor GTPBP2 (31). Ribosomal profiling of neurons from these mice showed a high incidence of stalled translation elongation complexes and increased GCN2-mediated eIF2α phosphorylation, yet showed no evidence for accumulation of an uncharged tRNA. This raised the intriguing possibility that GCN2 can also be activated by stalled ribosomes in addition to tRNA. Interestingly, GCN2 was most activated upon amino acid deprivation in cell lines with the most severe ribosome pausing (32).

If GCN2 can sense stalled ribosomes, it would suggest a functional relationship between GCN2 and the translation elongation machinery. The translation elongation cycle is primarily driven by the sequential actions of the GTPases eEF1A and eEF2. The GTPase activity of these translation factors is stimulated by a ribosomal protein complex known as the P-stalk that is part of the ribosomal GTPase-associated center (GAC) (33, 34). Short C-terminal tails (CTTs) that are present in each of the P-stalk proteins directly interact with GTPases and activate them (33–35). Amino acid deficiency can indirectly alter the translation cycle by reducing the availability of one or more acylated tRNAs, resulting in ribosome slowing or stalling. Whether or how GCN2 might monitor an altered translation cycle as a signal of nutrient starvation is unclear.

Here, we have reconstituted activation of human GCN2 in vitro using purified components. We show that human GCN2 interacts directly with ribosomes and by using a combination of hydrogen/deuterium exchange–mass spectrometry (HDX-MS) and truncation analysis, we have identified domain II of the ribosomal P-stalk protein uL10 [previously known as P0 (36)] as the principal GCN2 binding site. We have found that human GCN2 can be activated by purified ribosomes, the isolated recombinant P-stalk, and deacylated tRNA. Among these, ribosomes are the most potent activator. We show that the same CTTs, which are known to activate translational GTPases, also potently activate GCN2.

## Results

### Human GCN2 Is Activated by Ribosomes.

**Fig. 1.**
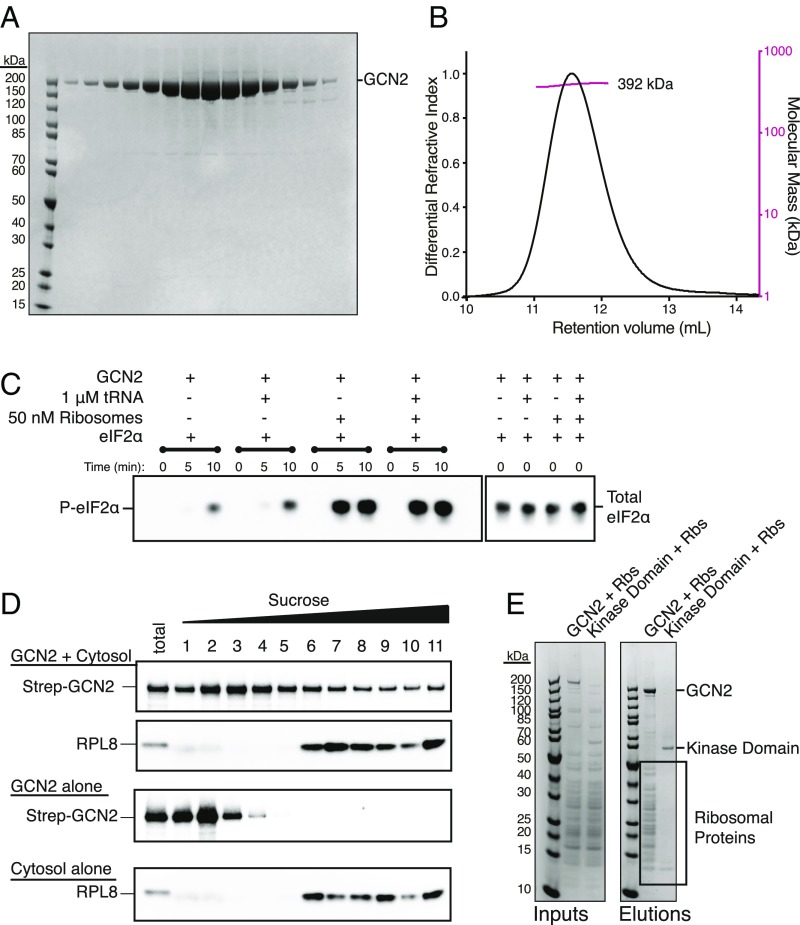
Purified ribosomes are a potent activator of GCN2. (*A*) SDS/PAGE analysis of gel filtration fractions for purified human GCN2 stained with Coomassie Blue. (*B*) Size-exclusion chromatography–multiangle light scattering (SEC-MALS) profile of GCN2. The molecular weight measured by light scattering was 392 kDa, consistent with a dimeric state. (*C*) eIF2α phosphorylation assay. The reactions were assembled and started by the addition of ATP. Samples were taken at 0, 5, and 10 min. The reactions were analyzed by SDS/PAGE and Western blotting with antibodies against phospho-eIF2α (P-eIF2α) and total eIF2α. (*D*) Migration of GCN2 through a sucrose gradient in the presence and absence of cytosol (rabbit reticulocyte lysate). The sample identities are shown on the *Left*. The reactions were incubated for 15 min before being run over a 10–50% sucrose gradient. Eleven fractions were collected and analyzed by SDS/PAGE and Western blotting with either an anti-StrepII tag or anti-RPL8 antibody. (*E*) Pulldown analysis of the GCN2–ribosome interaction. Either full-length GCN2 or the kinase domain only (residues 585–1,024) were incubated with purified ribosomes and the resultant complexes captured on StrepTactin resin. The beads were washed and the proteins eluted in sample buffer. The elutions were analyzed by SDS/PAGE and Coomassie staining. The bands corresponding to GCN2, the kinase domain, and ribosomal proteins are indicated on the *Right*.

To gain insight into how human GCN2 is activated, we expressed human GCN2 in *Sf9* cells and purified it to homogeneity (Fig. 1*A*). Size-exclusion chromatography–multiangle light scattering (SEC-MALS) calculated a molar mass of 392 kDa for the purified protein in solution, consistent with the theoretical mass of 384 kDa for a homodimer (Fig. 1*B*), which is in agreement with the oligomeric state of yeast GCN2 (25). GCN2’s main physiological substrate is eIF2α. Previous studies have shown that phosphorylation of eIF2α serine 51 is essential for its role in translational regulation downstream of GCN2 (37). We purified recombinant human eIF2α (*SI Appendix*, Fig. S1*A*) and assayed GCN2’s ability to phosphorylate it in vitro using a phospho-Ser51–specific antibody (Fig. 1*C*). GCN2 showed a detectable basal activity toward eIF2α in the absence of any putative activating factors.

The addition of total liver deacylated tRNA to 1 µM showed about a fivefold stimulation of eIF2α phosphorylation (Fig. 1*C*) with an EC_50_ of ∼500 nM (*SI Appendix*, Fig. S1*B*). Human GCN2 binds to deacylated tRNA with a *K*_D_ of ∼2 μM (*SI Appendix*, Fig. S1 *D*–*H*), which is similar to yeast GCN2 (13, 15). Increasing tRNA concentration to about 5× *K*_D_ resulted in an activation of about eightfold (*SI Appendix*, Fig. S1*B*). This tRNA interaction was abrogated by two mutations in the previously characterized tRNA-binding *m2* motif in the HisRS-like domain of GCN2 (F1143L R1144L, *SI Appendix*, Fig. S1 *D*–*H*), implying tRNA is binding to the *m2* motif as previously shown in yeast (15). Thus, GCN2 can phosphorylate eIF2α on Ser51, and this is stimulated approximately fivefold to eightfold by tRNA binding.

In marked contrast to 10 µM tRNA, 50 nM purified ribosomes dramatically increased the rate and extent of eIF2α phosphorylation (Fig. 1*C*). A titration indicated eIF2α phosphorylation by GCN2 is stimulated ∼20-fold by ribosomes, with an EC_50_ of ∼25 nM (*SI Appendix*, Fig. S1*C*). Deacylated tRNA did not further stimulate GCN2 beyond that seen with ribosomes alone. Thus, ribosomes show ∼20-fold greater potency and ∼3-fold higher maximal stimulation than observed with deacylated tRNA.

### GCN2 Directly Binds Ribosomes.

Given the remarkable activation of GCN2 by ribosomes in the purified, reconstituted system, we attempted to characterize the interaction between GCN2 and ribosomes. Recombinant, full-length, StrepII-tagged GCN2 was combined with rabbit reticulocyte lysate and fractionated by size using sucrose gradient sedimentation. A subset of GCN2 comigrated with the ribosomes in the cell lysate (Fig. 1*D*). In a parallel experiment, we found that purified ribosomes are captured by StrepII-tagged GCN2 immobilized on StrepTactin resin (Fig. 1*E*). In contrast, ribosomes were not recovered in complex with the GCN2 kinase domain alone (residues 585–1,024).

**Fig. 2.**
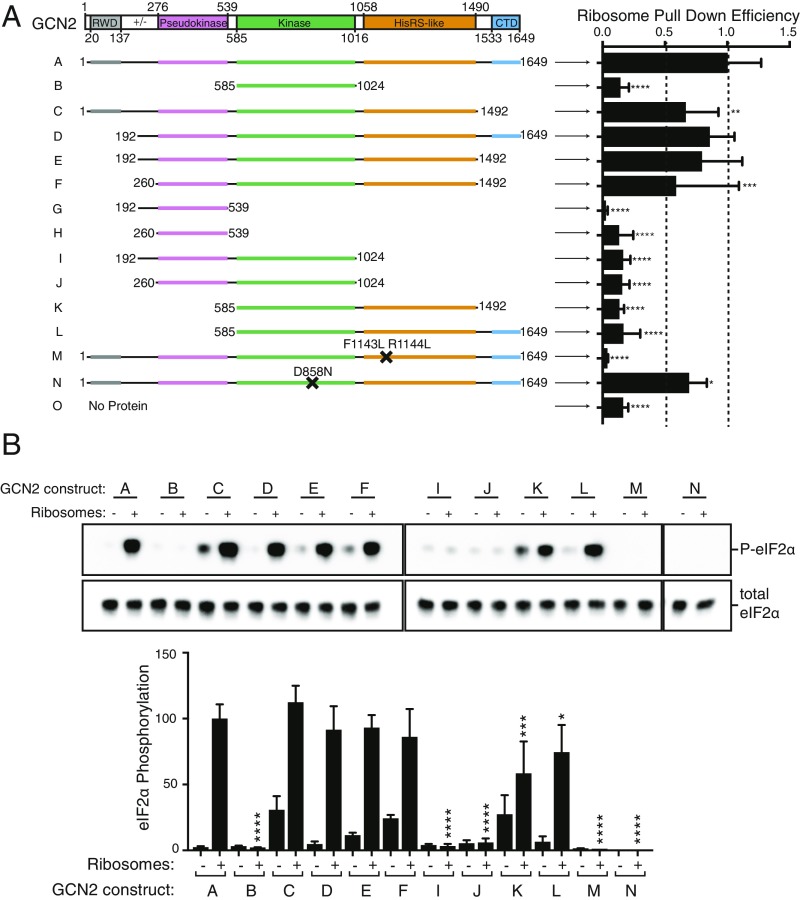
Several GCN2 domains contribute to ribosome binding and kinase activation. (*A*) Truncation analysis of the GCN2–ribosome interaction. The constructs were incubated with purified ribosomes, and the resultant complexes were captured on StrepTactin resin. The elutions were analyzed by SDS/PAGE and Coomassie staining. The intensities of three ribosomal proteins were quantified for each elution and normalized to the intensity of the bait protein. Three independent experiments were performed, and the means ± SDs are plotted. Statistical significance is shown by asterisks (****P < 0.0001). (*B*) The ability of each construct (at a concentration of 25 nM) to phosphorylate eIF2α was tested in the presence and absence of ribosomes (50 nM). The reactions were started by the addition of ATP/MgCl_2_ (0.5 mM/18.75 mM) and quenched after 5 min. The samples were analyzed by SDS/PAGE and Western blotting using antibodies against P-eIF2α and total eIF2α.

A series of GCN2 domain truncations and residue substitutions (Fig. 2*A* and *SI Appendix*, Fig. S2; full details in *SI Appendix*, Table S1) identified the domains of GCN2 that influence ribosome binding. Each recombinant protein was purified, immobilized on StrepTactin resin, and used in pulldown experiments with purified ribosomes. This analysis showed that three GCN2 domains are needed for maximal ribosome binding: the pseudokinase domain, the HisRS-like domain, and the CTD (Fig. 2*A* and *SI Appendix*, Fig. S2*B*). Comparing constructs D and L shows that the pseudokinase domain contributes to ribosome binding (Fig. 2*A*). However, the pseudokinase domain alone is not sufficient to bind ribosomes (constructs G and H) and additionally needs the HisRS-like domain. Indeed, each of the constructs lacking the HisRS-like domain (Fig. 2*A*, constructs I and J) showed only a very low ribosome binding efficiency. The previously characterized *m2* mutant in the HisRS-like domain (Fig. 2*A*, construct M, with human F1143L and R1144L mutations, equivalent to yeast Y1119 and R1120), which is unable to bind tRNA (15), also significantly decreases binding to ribosomes. A smaller, but detectable decrease in ribosome binding is seen when the CTD is deleted (construct C).

### Key Domains of GCN2 Required for Activation of eIF2α Phosphorylation by Ribosomes.

Using the in vitro eIF2α phosphorylation assay, we examined the ability of each construct that contains the kinase domain to phosphorylate eIF2α both in the presence and absence of ribosomes (Fig. 2*B* and *SI Appendix*, Fig. S2*C*). To detect even weak activation by the various GCN2 constructs, these single-point assays were carried out at 50 nM ribosomes, twice the EC_50_ for ribosomes. Full-length GCN2 (construct A) was able to phosphorylate eIF2α at a low level in the absence of ribosomes and was significantly stimulated by the addition of ribosomes. The eIF2α phosphorylation was due to the purified human GCN2, since the D858N point mutant that eliminates GCN2 kinase activity still bound ribosomes but eliminated eIF2α phosphorylation (construct N, Fig. 2 and *SI Appendix*, Fig. S2). The kinase domain alone (construct B) has similar basal level of eIF2α phosphorylation, but it was not stimulated by ribosomes. Inclusion of the HisRS-like domain (construct K) reinstated ribosomal activation. Other constructs lacking the HisRS-like domain (constructs I and J) phosphorylated eIF2α at a basal level but showed no increase in activity in the presence of ribosomes. The pseudokinase domain of GCN2 seems to modestly contribute to maximal ribosomal stimulation (comparison of constructs K and F, Fig. 2*B* and *SI Appendix*, Fig. S2*C*). For constructs K and L, which had greatly impaired binding to ribosomes but had only partial decrease in activity in single-point assays, we performed titrations with ribosomes that confirmed that these constructs are significantly less active than the wild-type enzyme (*SI Appendix*, Fig. S2*E*).

Two GCN2 regions appear to have a role in the autoinhibition of the kinase activity in the absence of ribosomes. The first is the GCN2 CTD. Upon deletion of the CTD (construct C), GCN2’s basal activity increased ∼20-fold (Fig. 2*B* and *SI Appendix*, Fig. S2*D*), indicating that the CTD has a key role in maintaining GCN2 in an inactive state. The charged linker that connects the N-terminal RWD domain and the pseudokinase domain has a minor role in GCN2 repression, as when this is deleted (comparing construct F with E), GCN2’s basal activity increases twofold (Fig. 2*B* and *SI Appendix*, Fig. S2*D*). These findings demonstrate that in human GCN2 the HisRS-like domain is critical for both ribosome binding and kinase activation. The CTD serves an autoinhibitory function to minimize basal activity, consistent with the autoinhibitory role of the CTD in yeast (38). The pseudokinase domain, although contributing to stable GCN2-ribosome interaction, is mostly dispensable for GCN2 activation by the ribosome.

### GCN2 Interacts with the P-Stalk of the Ribosome.

**Fig. 3.**
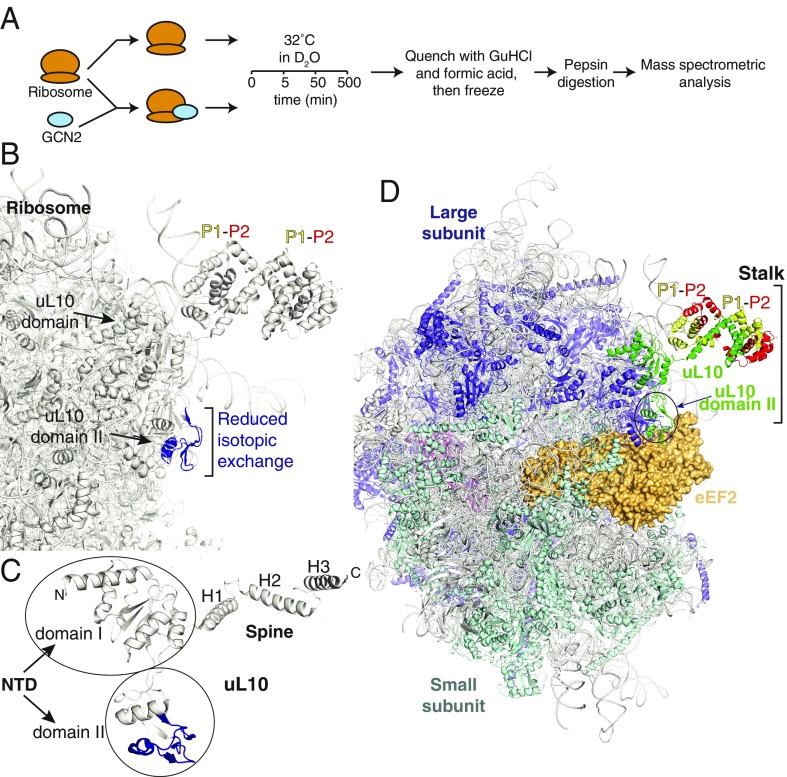
GCN2 binds to the ribosome via the P-stalk. (*A*) A schematic describing the HDX-MS experiment to determine the GCN2 binding site on the ribosome. Ribosomal samples in the presence or absence of GCN2 are incubated in deuterated buffer for 5, 50, or 500 min at 32 °C. The isotopic exchange is then quenched via a pH drop, and the samples are digested into peptides on a pepsin resin and analyzed by MS. The presence of GCN2 leads to a region with a significantly reduced rate of solvent exchange, indicative of a “binding footprint.” (*B*) Significant HDX-MS changes mapped onto the ribosomal structure (PDB ID 4V6X). The area with a reduced rate of isotopic exchange (residues 121–158 of uL10) is depicted in dark blue and indicated with a bracket. (*C*) Enlarged view of uL10, with HDX-MS changes as in *B*. (*D*) A view of the 80S ribosome with the ordered portion of the P-stalk shown and with bound eEF2 [from PDB ID 4V6X (47)]. The uL10 is shown as a green ribbon with two P1 (yellow)/P2 (red) heterodimers bound to the spine of uL10.

To study the structural basis of GCN2’s interaction with the intact ribosome, we made use of HDX-MS. Ribosomes purified from rabbit reticulocyte lysate were mixed with D_2_O-containing buffer solution at a final concentration of 0.5 μM ribosomes and incubated for varying lengths of times (5, 50, and 500 min) at 32 °C (Fig. 3*A*). Exchange was quenched, proteins were denatured, and the resulting solution was injected onto a pepsin column to cleave ribosomal proteins into peptides. Peptic peptides were separated on a reverse-phase column and analyzed using ion mobility separation MS. The same experiment was carried out in parallel for ribosomes mixed with 2.5 μM purified, recombinant GCN2. The differences in isotopic exchange between the two states (with and without GCN2) identified regions on the ribosome that were affected by GCN2. This method allowed us to make an unbiased map of the interaction of GCN2 with the ribosome.

This is the largest system yet to be examined with HDX-MS, covering 76 proteins digested into 1,070 peptides, representing 65% of the constituent sequences of the ribosomal proteins (Dataset S1). Despite the complexity, the results unambiguously identified a single continuous region of reduced isotopic exchange in the large ribosomal subunit protein uL10 (Fig. 3 *B* and *C* and *SI Appendix*, Fig. S3). This protein is part of the P-stalk, a heteropentameric complex that protrudes from the ribosome and forms part of the GAC where the translation factors eEF1A and eEF2 bind. Several peptides of uL10 showed a significantly reduced rate of deuterium uptake in the presence of excess GCN2 (*SI Appendix*, Fig. S3). While HDX-MS does not directly map protein–protein interactions, areas showing decreased isotopic exchange rates in the presence of a binding partner are likely to represent the binding site between the two proteins. The peptides identified as containing the interaction site with GCN2 are within residues 121–157 of uL10. This is part of domain II, a unique insertion in the N-terminal domain (NTD) of uL10 that is not present in bacteria. The insertion is on the surface of the ribosome, and it extends from the P-stalk toward the A site, where it can contact translational GTPases [e.g., eEF2 as shown in Fig. 3*D* (39)] on the 80S ribosome.

### The P-Stalk Interacts with Several Regions of GCN2.

**Fig. 4.**
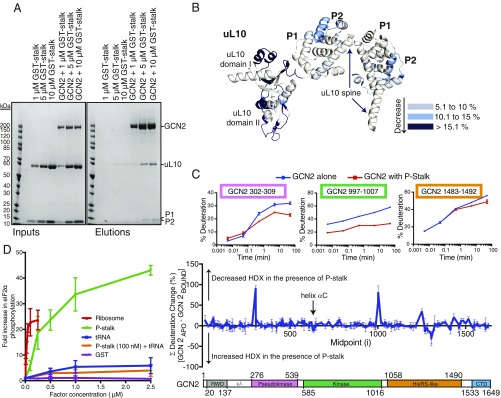
The isolated recombinant P-stalk binds GCN2. (*A*) Pulldowns showing that GCN2 can bind to the recombinant P-stalk. Increasing concentrations of the P-stalk were mixed with StrepTactin resin in the absence or presence of Strep-tagged GCN2, and the beads were then washed. Resultant complexes were eluted with sample buffer and analyzed by SDS/PAGE and Coomassie staining. The reaction inputs are shown on the *Left*, and the elutions are on the *Right*. (*B*) HDX-MS changes (summed over all time points) for the recombinant P-stalk in the presence of GCN2 are mapped on to the structure of the P-stalk (from 4V6X), colored according to the key on the *Right*. (*C*) HDX-MS changes for GCN2 in the presence of purified P-stalk. The percentage deuteration change was calculated by subtracting the percentage deuteration for each GCN2 peptide in the P-stalk–bound state from the same peptide in the apo state. The differences at each time point (0.3, 3, 30, 300, and 3,000 s) were summed for each peptide. The data shown in the *Middle* panel are the summed means of differences ± SDs (*n* = 3) plotted according to the peptide midpoint (*i*). A schematic of the GCN2 domain structure is given in the *Lower* panel. The *Upper* panel shows the time course for deuterium incorporation for three GCN2 peptides showing large differences in the rate of isotopic exchange in the presence of the P-stalk. (*D*) A comparison of stimulation of GCN2 by the P-stalk complex, ribosomes, and tRNA. Increasing concentrations of the various regulators were assayed for GCN2-mediated eIF2α phosphorylation. The reactions were analyzed by SDS/PAGE and Western blotting. Each titration included a control containing only GCN2 and eIF2α in the absence of any regulator, and the quantifications of the blots were normalized to this value. The data plotted are means ± SD for three independent experiments.

To validate the GCN2–uL10 interaction and investigate its functional relevance, we recombinantly expressed and purified the heteropentameric P-stalk complex consisting of uL10 bound to two P1–P2 heterodimers (*SI Appendix*, Fig. S4). P-stalk complexes were coexpressed in insect cells and purified under nondenaturing conditions. Pulldown assays showed that this P-stalk complex is able to bind to GCN2 immobilized on StrepTactin resin (Fig. 4*A*). HDX-MS analysis of the GCN2 interaction with the purified, recombinant P-stalk showed that six peptides in domain II of uL10 were protected upon addition of GCN2 (peptides 62–75, 76–89, 77–87, 121–137, 145–153, and 192–205; Fig. 4*B* and Dataset S2). Consistent with HDX-MS results with intact ribosomes, domain II peptides (121–137 and 145–153) show significant decrease in isotopic exchange upon interaction with GCN2. The isolated P-stalk additionally showed GCN2-dependent decreases in HDX in domain I of the NTD of uL10 that also contacts the 28S rRNA in the intact ribosome (Fig. 4*B*). Thus, by both pulldown and HDX-MS assays, the isolated P-stalk is sufficient for interaction with GCN2 in vitro.

HDX-MS of GCN2 in the absence versus presence of the ribosomal P-stalk showed a change in isotopic exchange for several regions of GCN2 (Fig. 4*C*). Reductions in the rate of exchange were seen in the pseudokinase domain (peptides 301–307, 302–309, and 453–459) and the HisRS-like domain, both of which were implicated in ribosome binding by truncation analysis (Fig. 2*A*). The HisRS-like domain showed both regions with reductions in isotopic exchange (peptides 1,248–1,254 and 1,483–1,492) and regions with increases in isotopic exchange (peptides covering the region 1,295–1,312), indicating that this domain may undergo complex conformational changes upon P-stalk binding. Significant difference in exchange were also seen for the CTD, where the two N-terminal helices show differences in exchange (*SI Appendix*, Fig. S5*B*). These helices form part of the dimer interface that is necessary for GCN2-mediated translation control (24).

Upon P-stalk binding, the kinase domain shows protection in the activation loop (peptides covering 866–903), a region near the C terminus of the domain (peptides covering 995–1,008) and in the insert that is not present in the yeast GCN2 kinase domain crystal structure (peptides in the region 662–794) (Fig. 4*C* and *SI Appendix*, Fig. S5). There is also an increase in isotopic exchange in a peptide (residues 630–639) in helix αC of the N-lobe of the kinase domain. This helix is one of the elements that is involved in the switch between the inactive and active forms of the yeast GCN2 (27, 28). In the inactive form of the kinase domain, in yeast GCN2 Glu-643 (equivalent to human Glu-636) in αC interacts with Arg-834 (equivalent to human Arg-847) to form a link between the N- and C-lobes of the kinase domain, which is disrupted upon GCN2 activation (28).

### The P-Stalk Is Sufficient to Activate GCN2 in Vitro.

Beyond a physical interaction, we found that the isolated P-stalk activates GCN2-mediated phosphorylation of eIF2α in the absence of the remainder of the ribosome (Fig. 4*D*). Titrating in increasing concentrations of the P-stalk complex gave an estimated EC_50_ for activation of 250 nM and about a 40-fold increase in activity at the highest concentration measured (2.5 μM). We also tested whether the P-stalk complex and tRNA had a synergistic effect on GCN2 by including a low concentration (100 nM) of the P-stalk and titrating in tRNA, but we saw no significant difference with respect to tRNA alone (Fig. 4*D* and *SI Appendix*, Fig. S6*A*), suggesting that there is no synergy. Among the three GCN2 activators we have tested, ribosomes are the most potent, followed by the isolated P-stalk, and then by deacylated tRNA (*SI Appendix*, Fig. S6*B*). The influence of the P-stalk on GCN2 is primarily an increase in specific activity, with no change in *K*_m_ (*SI Appendix*, Fig. S6*C*).

### The C-Terminal Acidic Motifs of the P-Stalk Subunits and uL10 Domain II Are Essential for Full GCN2 Activation.

**Fig. 5.**
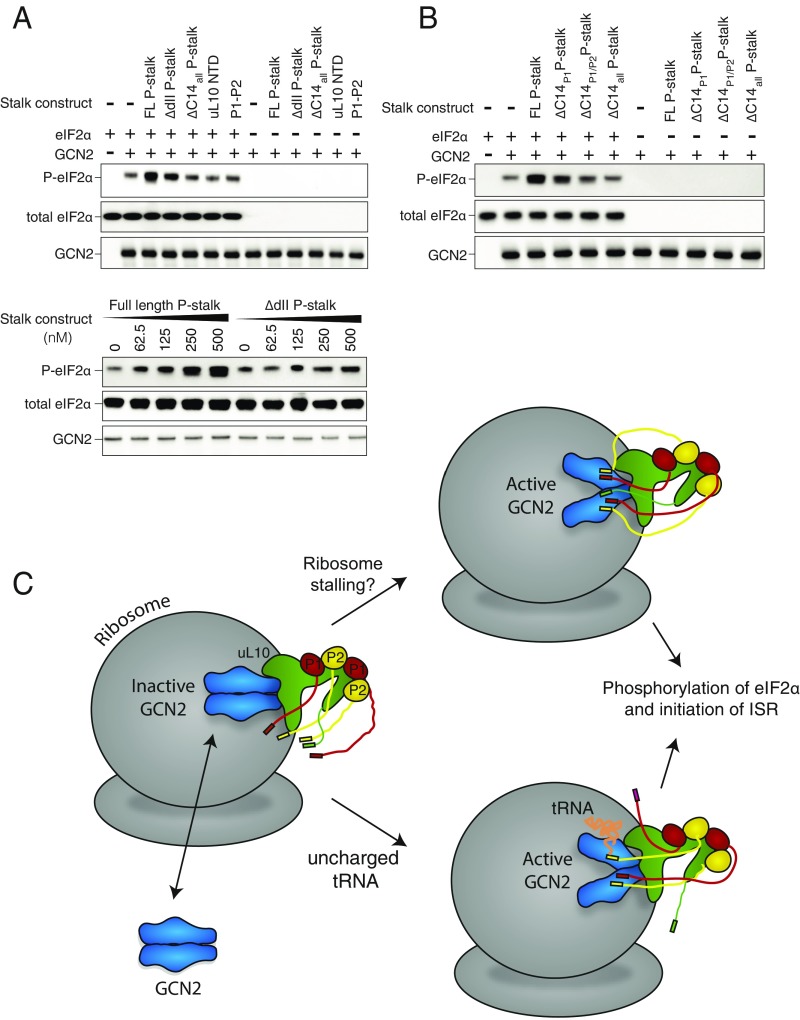
The domain II of u10 and the C-terminal tails (CTTs) of uL10, P1, and P2 are necessary for GCN2 activation. (*A*) The ability of different variants of the P-stalk to activate GCN2-mediated phosphorylation of eIF2α. (*Upper*) GCN2 and eIF2α were incubated with either the full-length (FL) P-stalk (uL10/P1/P2), ΔdII P-stalk [uL10(Δ111–183)/P1/P2], ΔC14_all_P-stalk [uL10(ΔC14)/P1(ΔC14)/P2(ΔC14)], uL10 NTD [uL10(1–208)], or P1/P2 (P1/P2). (*Lower*) GCN2 and eIF2α were incubated with a series of concentrations of either the full-length P-stalk (uL10/P1/P2) or ΔdII P-stalk [uL10(Δ111–183)/P1/P2]. The reactions were begun by the addition of ATP and then quenched after 5 min. The results were analyzed by SDS/PAGE and Western blotting with antibodies against the GCN2 Strep-tag, P-eIF2α, and total eIF2α. (*B*) The importance of the P1–P2 tails was tested by comparing eIF2α phosphorylation by GCN2 in the presence of either the full-length (FL) P-stalk, ΔC14_P1_ P-stalk [uL10/P1(ΔC14)/P2], ΔC14_P1/P2_ P-stalk [uL10/P1(ΔC14)/P2(ΔC14)], or ΔC14_all_P-stalk [uL10(ΔC14)/P1(ΔC14)/P2(ΔC14)]. (*C*) A schematic illustrating a model for GCN2 activation. The association of GCN2 with the P-stalk may be regulated, so that it occurs only in stress. Alternatively, GCN2 may associate with ribosomes via the uL10 domain II even in the basal state. During translation in amino acid-replete condition, the P-stalk CTTs interact with other components of the translation machinery, such as eEF1 and eEF2, and GCN2 remains in its inhibited state. When cells are starved of amino acids, ribosomes stall. Upon ribosomal stalling, the P-stalk CTTs may become free to activate GCN2. HDX-MS indicates that P-stalk binding by GCN2 results in conformational changes in multiple domains of GCN2, including the kinase, HisRS-like, and CTD domains. Uncharged tRNAs that accumulate under starvation also interact with GCN2. Activated GCN2 phosphorylates eIF2α to initiate the ISR.

Given that the P-stalk is a heteropentameric complex made up of three polypeptides, we expressed subcomplexes and variants of the P-stalk to determine which components are necessary for the activation of GCN2 (*SI Appendix*, Fig. S4). In contrast to the full-length P-stalk, the uL10 NTD did not activate GCN2 (Fig. 5*A*). Because HDX-MS indicated binding of GCN2 to domain II of uL10, we expressed the full P-stalk with domain II of uL10 deleted (ΔdII P-stalk, residues 111–183 of uL10 deleted). This P-stalk variant showed a greatly diminished activation of GCN2 (Fig. 5*A*). It has been proposed that P1 and P2 are sufficient for activation of GCN2 (16). However, our results show that, on its own, the human P1/P2 heterodimer did not activate GCN2 (Fig. 5*A*), suggesting that all three proteins of the P-stalk are required for activation. The C termini of uL10, P1, and P2 all contain a conserved 14-residue motif [SEESD(D/E)DMGFGLFD] that is implicated in activation of translational GTPases. Our HDX-MS analysis shows that the CTT of uL10 and a peptide (residues 73–93) adjacent to the CTT of P2 have significant protection in the presence of GCN2. To test whether these CTTs are also involved in GCN2 activation, we expressed the P-stalk with these 14 C-terminal residues deleted from each of the polypeptides (ΔC14_all_ P-stalk). Strikingly, ΔC14_all_ P-stalk was incapable of activating for GCN2 (Fig. 5*A*). Furthermore, deleting the CTTs from just P1 and P2 (leaving uL10 intact) dramatically decreases activation (Fig. 5*B*, ΔC14_P1/P2_ P-stalk). The extent of activation seems to be proportional to the number of CTTs available for interaction with GCN2, with activities in the order 5 CTT (WT) > 3 CTT (ΔC14_P1_) > 1 CTT (ΔC14_P1/P2_) > 0 CTT (ΔC14_all_) (Fig. 5*B*).

## Discussion

The role of GCN2 in the ISR pathway as a unique convergence point of nutrient availability and cell growth and proliferation has stirred interest in this kinase as a possible target for pharmaceutical development. Much of our current understanding of GCN2 has been derived primarily from in vivo studies in yeast. While aspects of this pathway may be well conserved in eukaryotes, there seem to be key differences in the way that GCN2 is activated in yeast and mammals. Furthermore, the interplay between GCN2 and protein translation means that it can be difficult to disentangle direct versus indirect mechanisms of control using in vivo analysis. For these reasons, we sought to develop an in vitro system to study the mechanistic basis of GCN2 regulation.

Consistent with a wide range of studies in cells, we find that deacylated tRNA activates human GCN2 toward its substrate eIF2α in vitro. In addition, we find that human GCN2 is even more potently stimulated by ribosomes and the purified ribosomal P-stalk complex. The degree of activation by ribosomes and P-stalk is higher than with deacylated tRNA, which does not achieve maximal stimulation of GCN2 even at a concentration five times its *K*_D_. We see no synergy in activation by P-stalk and deacylated tRNA, which may imply that these two activators operate either in parallel, in different cellular compartments or under different circumstances in vivo.

The in vivo activity of GCN2 has been previously linked to ribosomes in both yeast and mammalian systems (11, 20). However, a direct effect of ribosomes on GCN2 activity has not been demonstrated previously. Recently, Ishimura et al. (31) showed that GCN2 is activated in the brains of mice lacking a single isoacceptor tRNA and the putative ribosome rescue factor GTPBP2. Because these mice show widespread ribosomal stalling yet no change in deacylated tRNA levels, the authors proposed that a stalled ribosome might activate GCN2 in a tRNA-independent manner. The findings presented here are consistent with a model whereby GCN2 is receptive to activating signals when it is associated with the ribosome. Uncharged tRNAs may be one such signal, although currently there is no evidence that deacylated tRNAs can occupy the A site of eukaryotic ribosomes. The observations of Ishimura et al., whereby GCN2 is prominently activated under conditions in which ribosomes stall with no increase in uncharged tRNA, are easy to reconcile with our finding that human GCN2 physically interacts with ribosomes and is activated by P-stalk CTTs. These observations begin to provide a molecular framework for in vivo studies of mammalian GCN2.

As part of our analysis of ribosome-mediated GCN2 activation, we found that the previously characterized *m2* mutant in the HisRS-like domain cannot be activated by ribosomes in vitro. The homology of this domain to a tRNA synthetase, together with the inactivating *m2* mutant, was a major line of evidence implicating deacylated tRNA as a direct activator (13, 14). Our observation that tRNA-independent activation by ribosomes also critically depends on this motif suggests that results with this mutant may arise from a complex mechanism.

Our HDX-MS results showing that GCN2 binds to ribosomes via the P-stalk suggest a role for GCN2 as a factor monitoring ribosomal conformation or functional status. The P-stalk is adjacent to the A site of the ribosome and is thought to be involved primarily in the recruitment of translation factors during elongation (40). This would position GCN2 at the site of translational control. Previous results with yeast P-stalk components showed that P1 and P2 alone were sufficient for GCN2 activation (16), yet in contrast we find that human P1 and P2 proteins have no influence on GCN2 activity in the absence of uL10. However, we find that CTTs of P1 and P2 are critical for activating GCN2-mediated phosphorylation of eIF2α. Since HDX-MS indicates that the uL10 NTD is the predominant site of interaction with GCN2, our results imply that the P-stalk makes a bipartite interaction with GCN2. The isolated P-stalk fully activates GCN2 in vitro, but with an EC_50_ that is higher than for intact ribosomes. It may be that GCN2 makes other interactions with the ribosome that cannot be detected by HDX-MS, such as interacting directly with the 28S RNA.

The role of the P-stalk CTTs in activating GCN2 adds to the growing list of P-stalk CTT functions, as they are also important for growth (41), translation fidelity (42), interaction with translation factors (34, 35, 43), increasing GTPase activity of elongation factors in vitro (44), and interaction with ribosome inactivating proteins such as the ricin A-chain and trichosanthin (45). Both domain II and the CTTs are important for activation of eEF2 by archaeal P-stalk (46).

We do not know how P-stalk-mediated activation of GCN2 is regulated in cells (Fig. 5*C*). It could be that binding of human GCN2 to ribosomes in cells is regulated, so that it occurs only under stress. Alternatively, GCN2 could associate with ribosomes even under basal conditions by GCN2 binding to uL10 NTD, but activation by the P-stalk might occur only under stress conditions as the P-stalk CTTs become available. Upon cellular amino acid starvation, ribosome stalling could enable the CTTs to access and activate GCN2 through an unknown mechanism. Activated GCN2, either while still on the ribosome or after being released from it, would then phosphorylate eIF2. This observation that the P-stalk engages with both translational machinery and GCN2 creates further avenues for understanding the role of GCN2 in cellular homeostasis.

## Materials and Methods

### Protein Expression and Purification.

Recombinant StrepII-tagged GCN2 and GCN2 truncation variants as well as GST-tagged P-stalk proteins were cloned into baculovirus expression vectors and expressed in insect cells. Human eIF2α was expressed in bacterial cells. GCN2 constructs were purified by StrepTactin affinity chromatography, P-stalk constructs by glutathione affinity chromatography, and eIF2α by immobilized metal affinity chromatography. All constructs were further purified by anion exchange and gel filtration chromatography. Ribosomes were purified from rabbit reticulocyte lysate. Details of purifications are described in full in *SI Appendix*, *SI Materials and Methods*.

### Biophysical Analysis of GCN2.

The oligomeric state of GCN2 was determined by SEC-MALS. Binding of tRNA to GCN2 was quantitated by surface plasmon resonance, with the GCN2 covalently coupled to a CM5 chip (BiaCore), as described in full in *SI Appendix*, *SI Materials and Methods*.

### eIF2α Phosphorylation Assay.

eIF2α phosphorylation was detected by Western blotting with an anti–phospho-eIF2α antibody as described in *SI Appendix*, *SI Materials and Methods*.

### Binding Assays.

StrepII-tagged GCN2 constructs were bound to StrepTactin resin and ribosomal binding was detected by capturing purified ribosomes. Association with ribosomes was also shown by comigration of GCN2 with ribosomes in a sucrose gradient. For assay of P-stalk components binding to GCN2, purified GST-tagged P-stalk constructs were captured onto StrepTactin-bound GCN2. Details are described in *SI Appendix*, *SI Materials and Methods*.

### Deuterium Exchange Measurements.

Experiments were set up and analyzed as described fully in *SI Appendix*, *SI Materials and Methods*. Briefly, to detect GCN2 interactions with ribosomes, two reactions were prepared: one with 0.5 μM ribosomes alone and the other having 0.5 μM ribosomes and 2.5 μM GCN2. Both reactions were incubated on ice for 15 min, and then buffer with D_2_O was added to bring the D_2_O concentration to 78%, and samples were further incubated for 5, 50, and 500 min at 32 °C. Exchange was quenched, and samples were frozen in liquid nitrogen. Samples to analyze P-stalk complex/GCN2 interactions were similarly prepared, except deuteration was carried out for 0.3, 3, 30, 300, and 3,000 s. Samples were prepared for GCN2 alone, P-stalk complex alone, and two samples of GCN2 plus P-stalk: one sample contained 5 μM GCN2 and 15 μM P-stalk, while the other sample contained 15 μM GCN2 combined with 5 μM P-stalk.

## Supplementary Material

Supplementary File

Supplementary File

Supplementary File
