# Supplementary material for: Activation of GCN2 by the ribosomal P-stalk
Source: Proc Natl Acad Sci U S A. 2019 Feb 25;116(11):4946–54. doi: 10.1073/pnas.1813352116 (PMC6421407; doi:10.1073/pnas.1813352116)
Supplement: Supplementary File [file pnas.1813352116.sapp.pdf]

12/28/18 11:01:48 AM

PNAS
www.pnas.org

Supplementary Information for

Activation of GCN2 by the ribosomal P-stalk

Inglis, A. J., Masson, G. R., Shao, S., Perisic, O., McLaughlin, S. H., Hegde, R. S. and Williams, R. L.

Roger L Williams
Email: rlw@mrc-lmb.cam.ac.uk

This PDF file includes:

Supplementary text
Figs. S1 to S5
Table S1
Captions for additional data tables S2 to S3
References for SI reference citations

Other supplementary materials for this manuscript include the following:

Additional data tables S2 to S3

Supplementary Information Text

SI materials and methods

Baculoviruses for insect cell protein expression

www.pnas.org/cgi/doi/10.1073/pnas.1813352116

Baculoviruses for production of human GCN2 and human P-stalk complexes were produced in *Spodoptera frugiperda* (*Sf9*) cells. Cells were grown in Insect-Xpress Protein-Free medium (Lonza BioWhittaker, BE12-730Q) at 27 °C. Cells were transfected at a density of $0.5x10^6$ cells/mL with 2 – 4 µg of DNA using FuGENE HD Transfection Reagent (Promega E2311). Cells were grown at 27 ˚C for 5 days before the virus was harvested by centrifugation. The generated baculoviruses were used to produce proteins in Sf9 cells. Cells were collected, frozen in liquid N2 and stored at -80 °C.

Cloning of proteins

DNA encoding human GCN2 (uniprot ID: Q9P2K8) codon optimized for expression in insect cells was inserted into a pAceBac1 vector with an N-terminal twin StrepII tag followed by a TEV protease site. For the P-stalk components, standard In-Fusion cloning (TaKaRa) was used to create pAceBac1 single expression vectors encoding human stalk proteins ul10 (PCR-amplified from IMAGE:3463159, P1 (PCR-amplified from IMAGE:3343021) and P2 (PCR-amplified from IMAGE:4685028). The three expression cassettes for the P-stalk proteins were combined into a single pBIG1a vector using NEBuilder HiFi assembly (New England BioLabs E2621S) as previously described (1). The plasmids were integrated into EMBacY baculoviral DNA via Tn7 transposition (2), before the production of baculoviruses.

The following combinations of the human P-stalk proteins were created:

Plasmid AI73 (FL P-stalk) for GST-uL10/P1/P2
Plasmid AI69 (uL10-NTD) for GST-uL10(1-208)
Plasmid AI79 (ΔdII P-stalk) for GST-uL10(Δ111-183)/P1/P2
Plasmid AI82 (P1/P2) for P1/GST-P2
Plasmid AI86 ($\Delta C14_{all}$ P-stalk) for GST-uL10(1-303)/P1(1-100)/P2(1-101)
Plasmid AI87 ($\Delta C14_{P1/P2}$ P-stalk) for GST-uL10/P1(1-100)/P2(1-101)
Plasmid AI90 ($\Delta C14_{P1}$ P-stalk) for GST-uL10/P1(1-100)/P2 1.

A list of plasmids for all GCN2 constructs is given in Table S1.

DNA encoding full-length human eIF2α (NCBI reference number: NP_004085.1) was inserted into the vector pOPTH with an N-terminal His6 tag followed by a TEV protease site, before being expressed in *E. coli*.

Protein Purification

Purification of Strep-tagged proteins

GCN2 was expressed in *Sf9* cells by infecting cells at a density of 1.5x10^6 cells/mL with 15 mL of virus per 500 mL of cells. Cells were grown at 27 ˚C for 55 hours before being harvested and frozen in liquid nitrogen. The *Sf9* cell pellets were thawed and lysed in 100 mL lysis buffer A [20 mM tris(hydroxymethyl)aminomethane-HCl (Tris-HCl) pH 8.0 (room temperature), 150 mM NaCl, 5 % v/v glycerol, 2 mM β-mercaptoethanol, one cOmplete EDTA-free protease inhibitor tablet (Roche 04693132001)] per 2 L cells. Cells were lysed via a probe sonicator (Sonics Vibra-Cell VCX750) for 3 minutes (10 s on, 10 s off, 65 % power), then 6 U/mL Universal Nuclease (Pierce 88702) was added. The lysate was then subjected to centrifugation at 140,000*g* for 45 minutes at 4 ˚C. The resulting supernatant was passed through a 0.45 µM syringe filter (Millipore Sigma SE2M230I04) before being loaded onto a 5 mL StrepTrap HP column (GE Healthcare Life Sciences 28-9075-47), equilibrated in Strep-A buffer [20 mM Tris-HCl pH 8.0, 150 mM NaCl, 5 % v/v glycerol, 2 mM β-mercaptoethanol], at a flow rate of 2 mL/min. The column was washed with 50 mL Strep-A buffer and then the protein was eluted with 6 mM desthiobiotin in Strep-B buffer [20 mM Tris-HCl pH 8.0, 150 mM NaCl, 5 % v/v glycerol, 2 mM β-mercaptoethanol, 6 mM desthiobiotin (IBA 2-1000-005)]. The peak fractions were pooled and diluted 1:2 with Q_0 buffer [20 mM Tris-HCl pH 8.0, 5 % v/v glycerol, 2 mM β-mercaptoethanol] to reduce the salt concentration to 50 mM NaCl. The protein was then loaded onto a 5 mL HiTrap Q HP column (GE Healthcare Life Sciences 17-1153-01), equilibrated in Q-A buffer [20 mM Tris-HCl pH 8.0, 50 mM NaCl, 5 % v/v glycerol, 2 mM β-mercaptoethanol], at a flow rate of 3 mL/min. The column was washed with 50 mL Q-A buffer, and then the protein was eluted with a gradient of 0 – 100 % Q-B buffer [20 mM Tris-HCl pH 8.0, 1 M NaCl, 5 % v/v glycerol, 2 mM β-mercaptoethanol]. The peak fractions were pooled and concentrated to 1 mL using a 15 mL centrifugal filter (Amicon UFC901024 – UFC910024, depending on protein size) before injection onto a HiLoad 16/60 Superdex 200 column (GE Healthcare Life Sciences 17-1069-01), equilibrated with GF buffer [20 mM 4-(2-hydroxyethyl)-1-piperazineethanesulfonic acid (HEPES) pH 7.5, 150 mM NaCl, 2 mM tris(2-carboxyethyl)phosphine (TCEP)], at a flow rate of 1 mL/min. The peak fractions were

concentrated using a 15 mL centrifugal filter as above to 2 - 30 mg/mL before being aliquoted and frozen in liquid nitrogen.

Purification of P-stalk complexes

The P-stalk complexes were expressed in *Sf9* cells by infecting cells at a density of 1.5-2.0x10^6 cells/mL with 15 mL of virus per 500 mL of cells. Cells were grown at 27 ˚C for 72 hours before being harvested and frozen in liquid nitrogen. The *Sf9* cell pellets were thawed and lysed in 100 mL lysis buffer B [20 mM Tris-HCl pH 8.0 (room temperature), 100 mM NaCl, 5 % v/v glycerol, 5 mM dithiothreitol (DTT), one cOmplete EDTA-free protease inhibitor tablet (Roche 04693132001)] per 2 L cells. Cells were lysed via a probe sonicator (Sonics Vibra-Cell VCX750) for 3 minutes (10 s on, 10 s off, 65 % power), then 6 U/mL Universal Nuclease (Pierce 88702) was added. The lysate was then subjected to centrifugation at 140,000*g* for 45 minutes at 4 ˚C. The resulting supernatant was passed through a 0.45 µM syringe filter (Millipore Sigma SE2M230I04) before being mixed with 1 mL of Glutathione Sepharose 4B resin (GE Healthcare Life Sciences 17-0756-05), equilibrated in GST buffer [20 mM Tris-HCl pH 8.0 (room temperature), 100 mM NaCl, 5 % v/v glycerol, 5 mM DTT] for 60 minutes. The sample was then transferred to a BioRad gravity flow column, and the beads were washed with approximately 200 mL GST buffer. The protein was then eluted from the beads in 12 mL GST-E buffer [100 mM Tris-HCl pH 8.0 (room temperature), 100 mM NaCl, 15 mM glutathione] and then diluted 1:1 with Q_0 buffer to reduce the salt concentration to 50 mM NaCl. The protein was then loaded onto a 5 mL HiTrap Q HP column (GE Healthcare Life Sciences 17-1153-01), equilibrated in Q-A buffer, at a flow rate of 3 mL/min. The column was washed with up to 50 mL Q-A buffer, and then the protein was eluted with a gradient of 0 – 100 % Q-B buffer. The peak fractions were pooled and concentrated to 1 mL using a 15 mL centrifugal filter (Amicon UFC901024 – UFC910024, depending on protein size) before injection onto a HiLoad 16/60 Superdex 200 column (GE Healthcare Life Sciences 17-1069-01), equilibrated with GF buffer, at a flow rate of 1 mL/min. The peak fractions were concentrated using a 15 mL centrifugal filter as above to 3 – 5 mg/mL before being aliquoted and frozen in liquid nitrogen.

For the preparation of P-stalk complexes with the GST tag removed, the GST-tagged proteins were prepared as above, except that instead of eluting the protein from the

GSH resin by addition of glutathione, the GST tag was cleaved while the protein was bound on the GSH resin by addition of TEV protease (final TEV concentration of 0.1 mg/mL in the cleavage reaction). The resin was incubated with TEV for four hours or over night at 4 °C. Proteins were further purified on a HiTrap Q column as above and by gel filtration in GF buffer. Peak fractions were concentrated, aliquoted and frozen in liquid nitrogen.

Purification of eIF2α

The plasmid was transformed into chemically competent BL21 Star (DE3) cells, and cells were grown overnight before being inoculated to a 50 mL starter culture in 2xTY media containing 0.1 mg/mL Ampicillin. The starter culture was incubated at 37 ˚C for 90 minutes, then 12 mL starter culture was added to 4 x 1 L 2xTY media containing Ampicillin. Cultures were incubated at 37 ˚C until the optical density reached 1, and then protein expression was induced by the addition of 0.3 mM isopropyl β-D-1-thiogalactopyranoside (IPTG). Cells were grown for a further 3 hours at 37 ˚C before being harvested, washed with ice-cold phosphate-buffered saline and frozen in liquid nitrogen.

The bacterial cell pellets were lysed in 100 mL lysis buffer C [20 mM Tris-HCl pH 8.0, 100 mM NaCl, 5 % v/v glycerol, 2 mM β-mercaptoethanol, 0.5 mg/mL Lysozyme (Sigma L6876), 5 U/mL Universal Nuclease (Pierce 88702), one cOmplete EDTA-free protease inhibitor tablet (Roche 04693132001)] per 2 L cells. The cells were lysed using a probe sonicator (Sonics Vibra-Cell VCX750) for 5 minutes (10 s on, 10 s off, 65 % power), followed by centrifugation at 140,000*g* for 45 minutes at 4 ˚C. The supernatant was filtered using a 0.45 μM syringe filter (Millipore Sigma SE2M230I04) and then was loaded onto a 5 mL HisTrap HP column (GE Healthcare Life Sciences 17-5248-02), equilibrated in Ni-A buffer [20 mM Tris-HCl pH 8.0, 100 mM NaCl, 5 % v/v glycerol, 10 mM imidazole pH 8.0, 2 mM β-mercaptoethanol], at a flow rate of 3 mL/min. The column was then washed with 200 mL of Ni-A buffer, followed by the elution of the protein via a gradient of 0 – 100 % Ni-B buffer [20 mM Tris-HCl pH 8.0, 100 mM NaCl, 5 % v/v glycerol, 200 mM imidazole pH 8.0, 2 mM β-mercaptoethanol]. The peak fractions were then pooled and diluted 1:1 with Q_0 buffer as above, before being loaded onto a 5 mL HiTrap Q HP column equilibrated in Q-A buffer as above. The column was then washed with 50 mL Q-A buffer before the protein was eluted via

a 0 – 100 % gradient of Q-B buffer. Peak fractions were pooled and concentrated down to 1 mL using a 15 mL centrifugal filter (Amicon UFC901024). The sample was then injected onto a HiLoad 16/60 Superdex 75 column (GE Healthcare Life Sciences 17-1068-01), equilibrated with GF buffer at a flow rate of 1 mL/min. The peak fractions were concentrated using a 15 mL centrifugal filter (Amicon UFC901024) to approximately 8 mg/mL before being aliquoted and frozen in liquid nitrogen.

Biophysical Analysis of GCN2

Size Exclusion Chromatography-Multi Angle Light Scattering (SEC-MALS)

The SEC-MALS data were collected on a Heleos II instrument (Wyatt) coupled to an Optilab rEX online refractive index detector (Wyatt). The instrument was calibrated using Bovine Serum Albumin (BSA) (ThermoFisher Scientific 23209) as a standard. Purified proteins were diluted to 1 mg/mL in GF buffer and 100 µL volumes were injected onto a Superdex 200 10/30 column (equilibrated in GF buffer), with a flow rate of 0.5 mL/min.

Surface Plasmon Resonance

Surface Plasmon Resonance (SPR) data were collected using a BIAcore T200 instrument (GE Healthcare Life Sciences). GCN2 (at a concentration of 50 ng/µL) was directly immobilized onto a CM5 chip through amine coupling after activation of the chip with 1-ethyl-3-(3-dimethylaminopropyl)-carbodiimide hydrochloride (EDC) and N-hydroxysuccinimide (NHS). The protein was passed over the chip at 5 µL/min in a buffer containing of 10 mM sodium acetate pH 5.0. The analyte was deacylated tRNA (purified from porcine pancreatic cells using an established protocol (3)) at a range of concentrations prepared by a 1:1 serial dilution in RNC buffer [50 mM HEPES pH 7.5, 100 mM KOAc, 5 mM $MgAc_2$, 1 mM DTT]. The highest concentration was 40 µM. The tRNA was injected for 120 s in HEPES-buffered saline (HBS) buffer [20 mM HEPES pH 7.5, 150 mM NaCl] followed by a 600 s dissociation step. After each measurement, any remaining tRNA was dissocated with 2 M NaCl for 120 s. In parallel, a reference channel with no protein immobilised was injected with tRNA under the same conditions to check for non-specific binding. Data were doubly-referenced by subtraction of the reference channel data and from injections of buffer alone.

Purification of Ribosomes

A 5 mL aliquot of hemin-treated and nuclease-treated rabbit reticulocyte lysate (RRL) (Green Hectares, as described previously (4)) was thawed gently and immediately placed on ice. The RRL was then centrifuged at 539,994*g* for 40 minutes at 4 ˚C in a TLA100.3 rotor (Beckman Coulter 349490), and the supernatant was discarded. The pellet was washed with ribosomal wash buffer (RWB) [20 mM Hepes pH 7.5, 100 mM KOAc, 1.5 mM $MgAc_2$, 0.1 mM ethylenediaminetetraacetic acid (EDTA) pH 7.4), 1 mM DTT], and then resuspended in RNC buffer using a wide bore pipette tip. The sample was then transferred to a 5 mL glass Dounce tissue grinder and homogenised. Once entirely resuspended, the concentrations of KOAc and $MgAc_2$ were increased to a final concentration of 750 mM and 15 mM respectively. The sample was then layered over a 1 mL sucrose cushion [20 mM Hepes pH 7.5, 750 mM KOAc, 15 mM $MgAc_2$, 0.1 mM EDTA pH 7.4, 1 mM DTT, 1 M sucrose] and centrifuged at 539,994*g* for 1 hour at 4 ˚C in a TLA100.3 rotor (Beckman Coulter 349490). The supernatant was discarded and the pellet washed with RNC buffer. The pellet was then resuspended in RNC buffer as before and homogenised in a 1 mL glass Dounce tissue grinder, before being aliquoted and frozen in liquid nitrogen.

eIF2α Phosphorylation Assay

Due to problems with eIF2α associating with the reaction tubes, all eIF2α phosphorylation reactions were performed in tubes that had been previously coated in BSA according to the following protocol. 1 mL of a 100 mg/mL solution of BSA (Fisher Scientific BP1605) was dispensed into 1.5 mL eppendorf tubes and the tubes were left for 12 hours. The BSA was then aspirated, and the tubes twice washed with 1 mL phosphate-buffered saline. All liquid was then removed, and the tubes were left to dry thoroughly.

The reactions were performed in assay buffer [50 mM Hepes pH 7.4, 100 mM KOAc, 5 mM $MgAc_2$, 1 mM DTT, 6.25 mM β-glycerophosphate (Sigma 50020)] with GCN2 at a final concentration of 25 nM, eIF2α at a final concentration of 250 nM, ± 1 µM tRNA, ± 50 nM ribosomes, ± 250 nM P-stalk variants (unless otherwise indicated). Each reaction also contained 1 mg/mL bovine serum albumin (Fisher Scientific BP1605). The reactions were started upon the addition of an ATP mix [final concentrations 0.5 mM MgATP pH 8.0, 18.75 mM $MgCl_2$] and the transfer from ice to 32 ˚C. Samples were taken at 0, 5 and 10 minutes and quenched by the 1:1 addition of

2X LDS sample buffer. All samples were boiled for 5 minutes before being run on a 4-12 % Bis-Tris Protein Gel (NuPAGE NP0329). The proteins were then transferred to a nitrocellulose membrane using the iBlot Gel Transfer Device (Invitrogen) in combination with iBlot Transfer Stacks (Invitrogen IB301002) or onto a PVDF membrane, using a Trans-Blot Turbo Transfer System (Bio-Rad 170-4157). The membrane was blocked for 1 hour in 5 % BSA (ThermoFisher Scientific BP1605) in TBST buffer and washed three times in TBST buffer. The membrane was then incubated with the primary antibody [Cell Signalling #9721 (1:1000 or 1:500, depending on the lot) for phospho-eIF2α; Santa Cruz Biotech sc-133132 (1:1000) for total eIF2α; abcam 76949 (1:1000) for Strep-tagged GCN2] for 1 hour, then washed three times in TBST before being incubated with the secondary antibody [Cell Signalling #7074 (1:5000) for phospho-eIF2α and Strep-tagged GCN2; Pierce 31430 (1:10,000 dilution) for total eIF2α] for 1 hour and subsequently washed three more times in TBST. (All antibodies were diluted at the indicated dilutions in 5 % BSA in TBST.) Finally, the membranes were incubated with 2 mL SuperSignal West Pico PLUS chemiluminescent substrate (ThermoFisher Scientific 34577) and exposed using a ChemiDoc Touch Imaging System (Bio-Rad).

Binding Assays

Ribosomal Co-migration Assays

Reactions were carried out in 50 µL aliquots that contained 100 nM Strep-II-tagged proteins and 35 µL rabbit reticulocyte lysate (RRL, Green Hectares) in RNC buffer. Reactions were incubated at 32 ˚C for 15 minutes. 200 µL gradients containing 10 – 50 % sucrose in RNC buffer were assembled in five 40 µL steps in polycarbonate centrifuge tubes (Beckman Coulter 343775) and left to sit on ice for 30 minutes. An aliquot of 20 µL of the reaction was loaded onto the top of the gradients, and then the gradients were subjected to centrifugation at 259,000*g* in a TLS-55 rotor (Beckman Coulter 346936) for 30 minutes at 4 ˚C, with an acceleration of 9 and a deceleration of 0. Eleven 20 µL fractions were manually collected from the top of the gradient and mixed with 20 µL 2X LDS sample buffer (NuPAGE NP0008). Samples were run on a 4-12 % Bis-Tris Protein Gel (NuPAGE NP0329), and then were Western blotted as described above. The primary antibodies were Anti-Strep-tag II antibody (abcam 76949, 1:1000 dilution) or Anti-RPL8 (abcam ab169538, 1:1000 dilution) and the secondary antibody was Anti-Rabbit (Cell Signalling #7074, 1:5000 dilution).

Ribosomal Pull-Down Assays

Aliquots containing 500 µL reactions including 100 nM Strep-tagged proteins and 100 nM ribosomes were assembled in RNC buffer, and then incubated at 32 ˚C for 15 minutes. 20 µL of StrepTactin Sepharose High Performance resin (GE Healthcare Life Sciences 28-9355-99), equilibrated in RNC-Wash buffer [RNC buffer + 0.1 % Triton X-100 (Sigma T8787)] was added to each reaction and the reactions were rotated at 4 ˚C for 90 minutes. The beads were then sedimented by centrifugation at 500*g* for 1 minute at 4 ˚C, before the supernatant was removed and the beads were resuspended in 1 mL RNC-Wash buffer. This washing step was repeated seven times in total and the reactions were transferred to fresh tubes. All solution was then aspirated and proteins were eluted from the beads by the addition of 18 µL 2X LDS sample buffer (NuPAGE NP0008). Samples were then run on a 4-12 % Bis-Tris Protein Gel (NuPAGE NP0329) and stained using InstantBlue Protein Stain (Expedeon 1SB1L).

GCN2-Stalk Pull-Down Assays

Pull downs were carried out by mixing Strep-tagged GCN2 at 1 µM with the P-stalk complex at 1, 5 or 10 µM in RNC buffer, with a final reaction volume of 40 µL. The reactions were incubated at 32 ˚C for 15 minutes, and then 10 µL StrepTactin Sepharose High Performance resin (GE Healthcare Life Sciences 28-9355-99) (equilibrated in RNC buffer) was added to each reaction. The reactions were rotated at 4 ˚C for 90 minutes. The beads were then sedimented by centrifugation at 500*g* for 1 minute at 4 ˚C, before the supernatant was removed and the beads were resuspended in 1 mL RNC-Wash buffer. This washing step was repeated three times in total and the reactions transferred to fresh tubes. All solution was then aspirated and the proteins eluted from the beads through the addition of 18 µL 2X LDS sample buffer (NuPAGE NP0008). Samples were then run on a 4-12 % Bis-Tris Protein Gel (NuPAGE NP0329) and stained using InstantBlue Protein Stain (Expedeon 1SB1L).

Hydrogen/Deuterium Exchange-Mass Spectrometry

Preparation of Ribosome-GCN2 samples

Hydrogen-Deuterium exchange-mass spectrometry (HDX-MS) samples were prepared from two reactions: one consisting of ribosomes alone at 0.5 µM, and a second consisting of ribosomes at 0.5 µM and GCN2 at 2.5 µM. All reactions were assembled

in RNC buffer. Both reactions were incubated on ice for 15 minutes and then aliquoted into 10 µL aliquots. To each reaction, 40 µL of deuterated RNC buffer [50 mM HEPES pH 7.5, 100 mM KOAc, 5 mM $MgAc_2$, 94.8 % D_2O (Acros Organics 7789-20)] was added and the reactions mixed rapidly, producing a final D_2O concentration of 75.84 %. Three incubation times were tested in triplicate: 5 minutes, 50 minutes and 500 minutes, all at 32 ˚C. To ensure the prolonged 500 minute time point was not having a detrimental effect on the structure of the ribosome, a pulse experiment was also performed in which a 10 µL protein aliquot was incubated at 32 ˚C for 495 minutes before 40 µL deuterated RNC buffer was added for 5 minutes. All reactions were quenched via the addition of 20 µL ice-cold Quench buffer [5 M GdCl, 8.4 % formic acid], followed by freezing in liquid nitrogen.

Preparation of GCN2-Stalk samples

Samples for HDX-MS were prepared from four reactions. For analysis of exchange for GCN2 peptides, there were two reactions: one consisting of GCN2 alone at 5 µM, and one consisting of GCN2 at 5 µM and the P-stalk complex at 15 µM. For analysis of exchange for P-stalk complex peptides, there were also two reactions: one consisting of P-stalk complex alone at 5 µM, and one consisting of P-stalk complex at 5 µM and the GCN2 at 15 µM. All reactions were assembled in RNC buffer. The reactions were incubated on ice for 15 minutes and then aliquoted into 10 µL aliquots. 40 µL of deuterated RNC buffer (described above) was added to each reaction and then mixed rapidly, producing a final D_2O concentration of 75.84 %. Five incubation times were tested in triplicate: 0.3 seconds, 3 seconds, 30 seconds, 300 seconds and 3000 seconds. Due to practical issues with producing a 0.3 second time point, these samples were prepared at 0 ˚C, reducing the rate of deuteration by approximately 10 fold (5). All the reactions were quenched via the addition of 20 µL ice-cold Quench buffer [5 M GdCl, 8.4 % formic acid], followed by snap freezing in liquid nitrogen.

HDX LC MS/MS Sample Analysis

Each sample was thawed and injected onto the M-Class Acquity UPLC with HDX technology (Waters) kept at 0.1 ˚C. The proteins were digested on an Enzymate Pepsin Column (Waters, 186007233) at 15 ˚C for two minutes. The peptides were then eluted from the column onto an Acquity UPLC BEH C18 VanGuard Pre-column (Waters, 186003975), equilibrated in Pepsin-A buffer [0.1 % formic acid]. The pre-column was

eluted into an Acquity UPLC BEH C18 column (Waters, 186002346), using a 3 - 43 % gradient of Pepsin-B buffer [0.1 % formic acid, 99.9 % acetonitrile] over 22 minutes. Data were collected on a Waters Synapt G2-Si in HD-MS^e (with ion mobility separation) mode with an electrospray ionisation source, and scanned with a 50 to 2000 m/z range. The spray voltage was 3.0 kV.

Quantification and Statistical Analysis

Size-Exclusion Chromatography-Multi Angle Light Scattering

The protein concentration was measured using the refractive index (RI), on the basis of 1 mg/mL protein having a ΔRI of 0.186 mL/g, and the observed scattering intensities were used to calculate the absolute molecular mass using the ASTRA software package (Wyatt).

Surface Plasmon Resonance

The data were analysed in Prism, and the buffer response from the reference channel was subtracted from the data. The K_D was calculated using both kinetic and equilibrium methods.

eIF2α Phosphorylation Assay

The blots were quantified using ImageJ. Band intensities were measured, and then the background values subtracted. The values were then normalised to the amount of eIF2α phosphorylation by the full-length protein in the presence of ribosomes. Values plotted are the means ± the standard deviations of three independent experiments.

Ribosome Pull-Down Assays

Three ribosomal protein bands were quantified across the gel using Image Lab 2 (BioRad) and normalized to the pull down efficiency of the full-length GCN2. Values plotted are the means ± the standard deviations of these three bands, across three independent experiments (giving nine values in total).

Hydrogen/Deuterium Exchange-Mass Spectrometry

Peptides were identified using ProteinLynx Global Server (Waters, 720001408EN). Three replicates of non-deuterated ribosomes were analysed by HD-MS^e (5). To qualify for further analysis, peptides had to fulfil the following criteria:

1. Minimum intensity of 5,000 counts
2. Maximum length of 25 amino acids
3. Minimum number of products of three
4. Minimum number of products per amino acid of 0.05
5. Maximum mass error of 10 ppm

For the P-stalk/GCN2 dataset, the peptide length criterion was relaxed to allow for the detection of the C-terminal termini of the P-stalk that typically presented as longer petides (35 amino-acids or greater). Peptides that fulfilled the stated criteria were imported into DynamX (Waters, 720005145en) for data analysis. All data was subjected to automated data processing by DynamX, followed by manual inspection of the data. Peptides that showed an unacceptably low signal-to-noise ratio precluding robust analysis were eliminated at this stage. Following this analysis, changes in the deuteration profile for each peptide upon the addition of excess binding partners were statistically analysed to determine the level of significance for the dataset. Peptides that showed changes greater than this threshold were therefore considered to be significantly affected by the presence of the binding partner.

Data and Software availability

Hydrogen/Deuterium Exchange-Mass Spectrometry

The full HDX-MS datasets are included as Supplementary datasets. The full dataset for the GCN2-Ribosome interaction is included as Dataset S1. The full dataset for the GCN2-P-stalk interaction is included as Dataset S2.

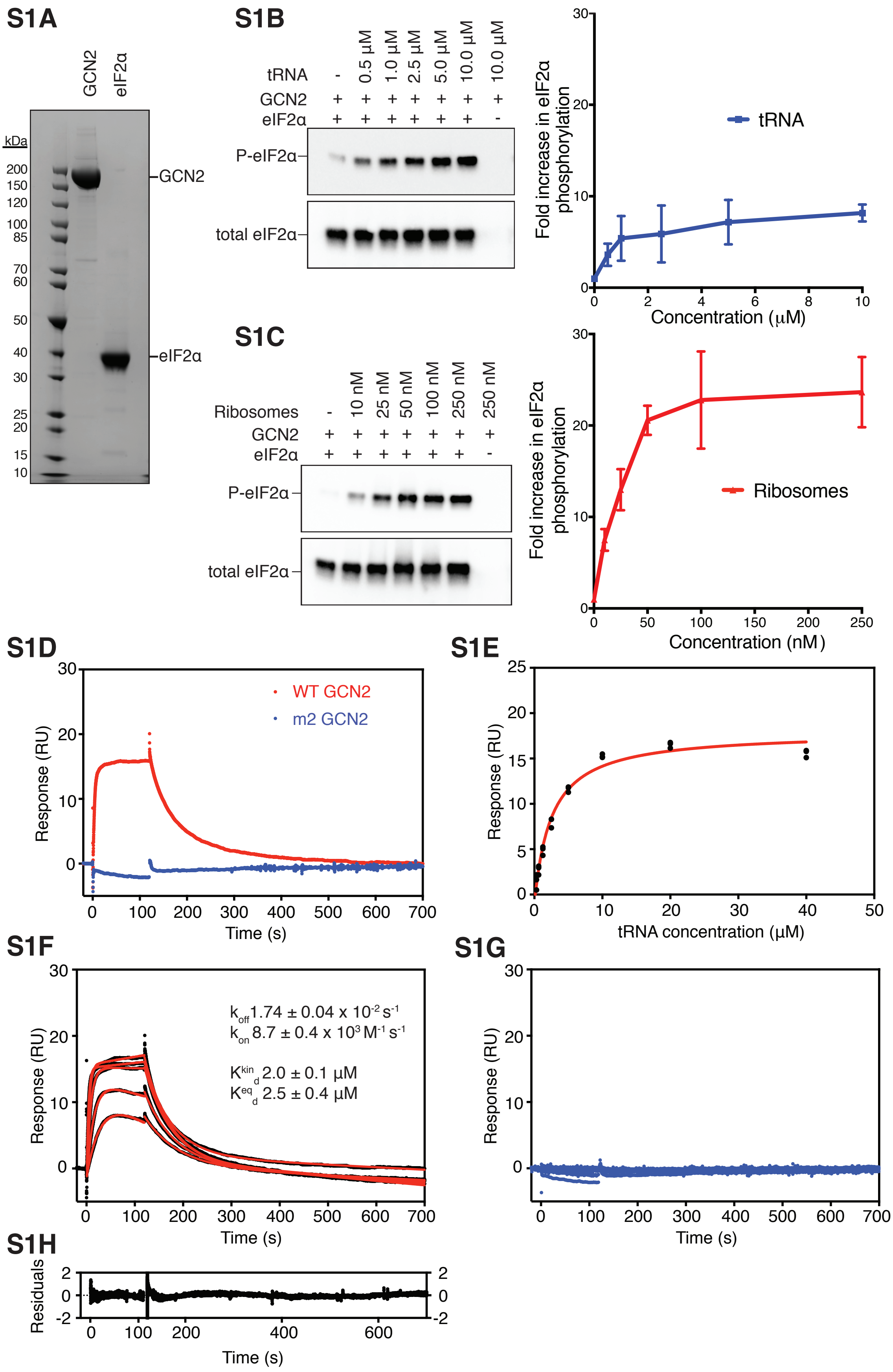
S1A
GCN2
eIF2α
kDa
200
150
120
100
85
70
60
50
40
30
25
20
15
10
GCN2
eIF2α
S1B
tRNA - 0.5 µM 1.0 µM 2.5 µM 5.0 µM 10.0 µM 10.0 µM
GCN2 + + + + + + +
eIF2α + + + + + + -
P-eIF2α
total eIF2α
Fold increase in eIF2α phosphorylation
tRNA
Concentration (µM)
S1C
Ribosomes - 10 nM 25 nM 50 nM 100 nM 250 nM 250 nM
GCN2 + + + + + + +
eIF2α + + + + + + -
P-eIF2α
total eIF2α
Fold increase in eIF2α phosphorylation
Ribosomes
Concentration (nM)
S1D
WT GCN2
m2 GCN2
Response (RU)
Time (s)
S1E
Response (RU)
tRNA concentration (µM)
S1F
k_{off} 1.74 ± 0.04 x 10^{-2} s^{-1}
k_{on} 8.7 ± 0.4 x 10^{3} M^{-1} s^{-1}
K^{kin}_{d} 2.0 ± 0.1 µM
K^{eq}_{d} 2.5 ± 0.4 µM
Response (RU)
Time (s)
S1G
Response (RU)
Time (s)
S1H
Residuals
Time (s)

Fig. S1. Ribosomes are a more potent activator of GCN2 than deacylated tRNA.

A. Coomassie stained SDS-PAGE gel showing the final purity of human GCN2 and eIF2α.

B. Phosphorylation of eIF2α by GCN2 in response to increasing amounts of deacylated tRNA (from 0 - 10 μM). The reactions were assembled and begun by the addition of ATP. The reactions were quenched by the addition of 1 volume of sample buffer after 5 minutes and were then analysed by SDS-PAGE and Western blotting, using antibodies against phospho-eIF2α and total eIF2α. A quantification of three independent experiments is shown on the right. The means ± the standard deviations are plotted.

C. Phosphorylation of eIF2α by GCN2 in response to increasing amounts of purified ribosomes (from 0 - 250 nM), analysed as for panel B.

D. The SPR response when 40 μM deacylated tRNA was flowed over a chip with immobilised wild-type (red trace) or *m2* mutant GCN2 (F1143L R1144L) (blue trace). Each sample was measured in two independent experiments.

E. The SPR responses upon flowing increasing concentrations of deacylated tRNA over a chip with immobilized wild-type GCN2.

F. The blank-corrected SPR responses for each deacylated tRNA concentration flowed over a chip with immobilized GCN2. The red lines indicate the fit for each response. The kinetic rate constants, kinetic K_d and the equilibrium K_d are shown on the graph.

G. The SPR responses upon flowing increasing concentrations of deacylated tRNA over a chip with immobilised *m2* mutant GCN2.

H. The residuals showing the deviations between the experimental and fits to the data.

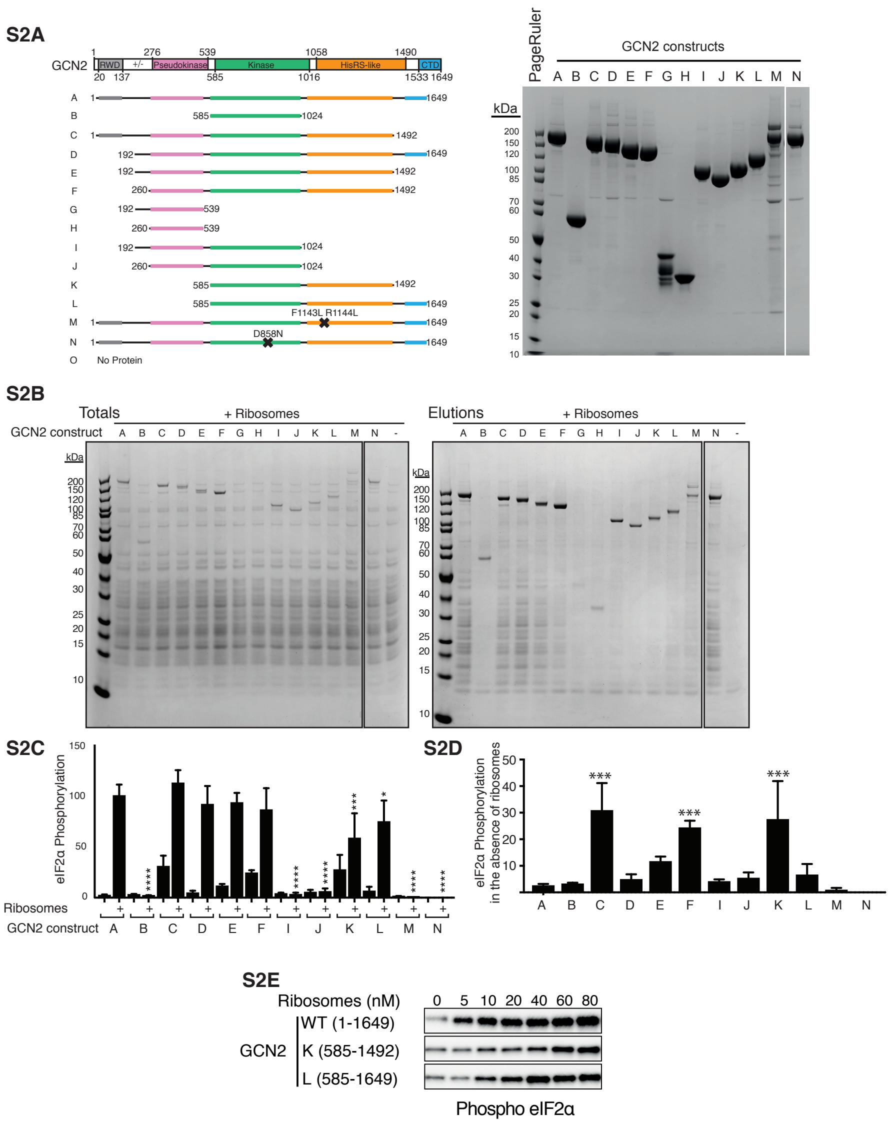

Fig. S2. Truncation analysis of the role of the GCN2 domains in phosphorylating eIF2α.

A. A schematic diagram illustrating each GCN2 construct used (left panel). Coomassie stained SDS-PAGE showing purified GCN2 constructs (right panel).

B. Pull-down reactions were assembled containing 100 nM of each StrepII-tagged GCN2 construct and 100 nM of purified ribosomes, and the reactions were incubated at 32 ˚C for 15 minutes. The complexes were captured on StrepTactin resin, and the resin was then washed. The proteins were eluted from the resin in sample buffer, and analysed by SDS-PAGE and Coomassie staining. The reaction inputs are shown on the left-hand gel and the elutions on the right-hand gel.

C. The ability of each construct to phosphorylate eIF2α was tested in the presence and absence of ribosomes. The samples were analysed by SDS-PAGE and Western blotting against P-eIF2α and total eIF2α. The quantifications were normalised according to the

amount of total eIF2α in each reaction, and then scaled to eIF2α phosphorylation by full-length GCN2 in the presence of ribosomes. The data plotted are the means ± the standard deviations for three independent experiments. Statistical significance is shown by asterisks (**** $P < 0.0001$), and is given for samples in the presence of ribosomes in comparison to construct A.

D. A zoomed-in representation of the ability of each construct to phosphorylate eIF2α in the absence of ribosomes. Statistical significance is shown by asterisks (**** $P < 0.0001$), and is given for samples in the absence of ribosomes in comparison to construct A.

E. eIF2α phosphorylation activity of wild-type GCN2 and truncation variants K and L in the presence of the increasing concentrations of ribosomes. Both K and L constructs have a higher EC50 for ribosomes when compared to wild-type GCN2.

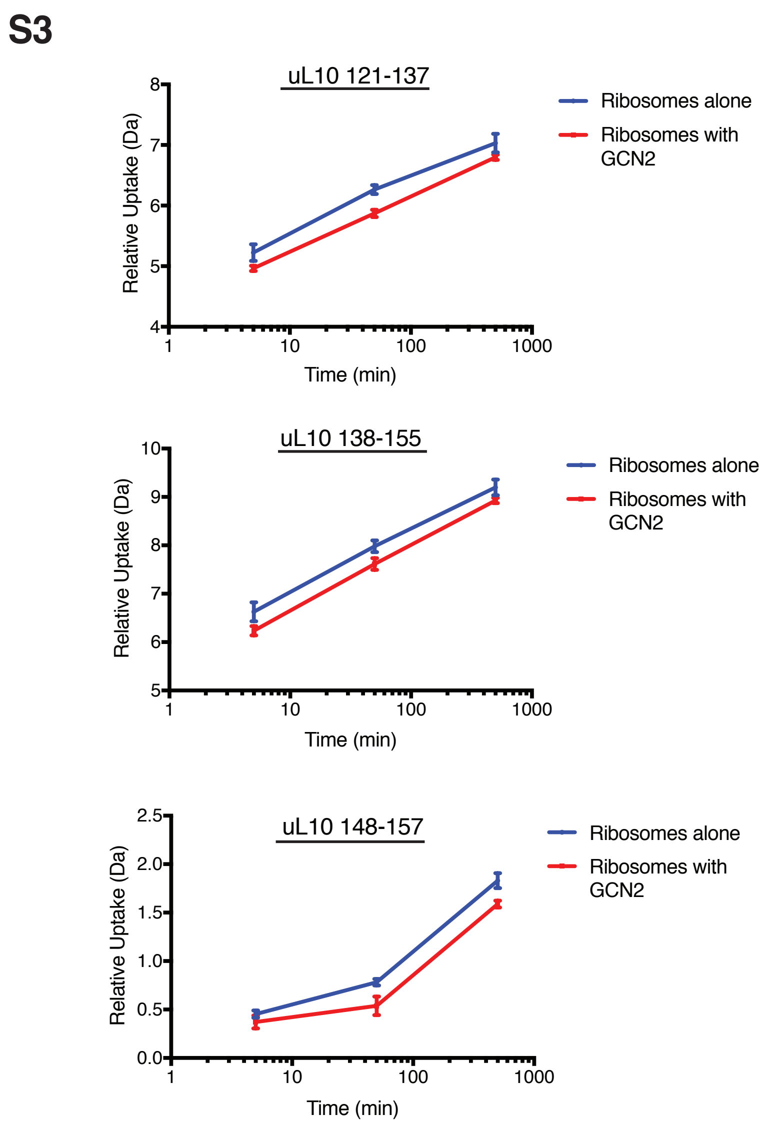

Fig. S3. Deuterium uptake plots for the ribosomal uL10 peptides in the presence and absence of GCN2.

The deuterium uptake plots for ribosomal peptides showing significant changes in the rate of deuterium uptake in the presence of GCN2 are shown. The data for ribosomes in the apo state are shown in blue, and ribosomes in the presence of GCN2 in red.

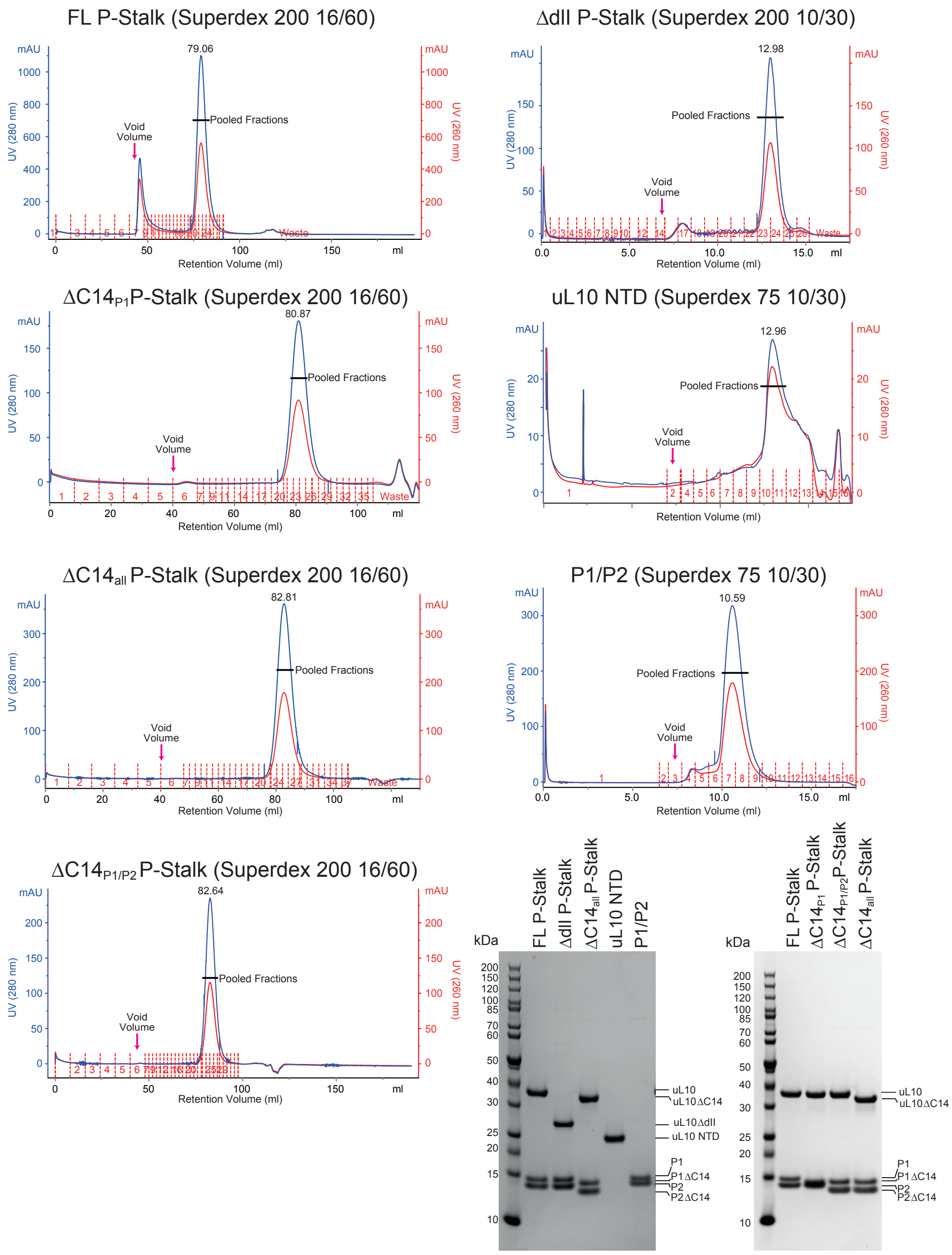

Fig. S4. Purified human ribosomal P-stalk variants.

Coomassie stained SDS-PAGE and gel filtration profiles showing the purity and integrity of the P-stalk complexes.

S5A Human GCN2 kinase domain colored by HDX differences

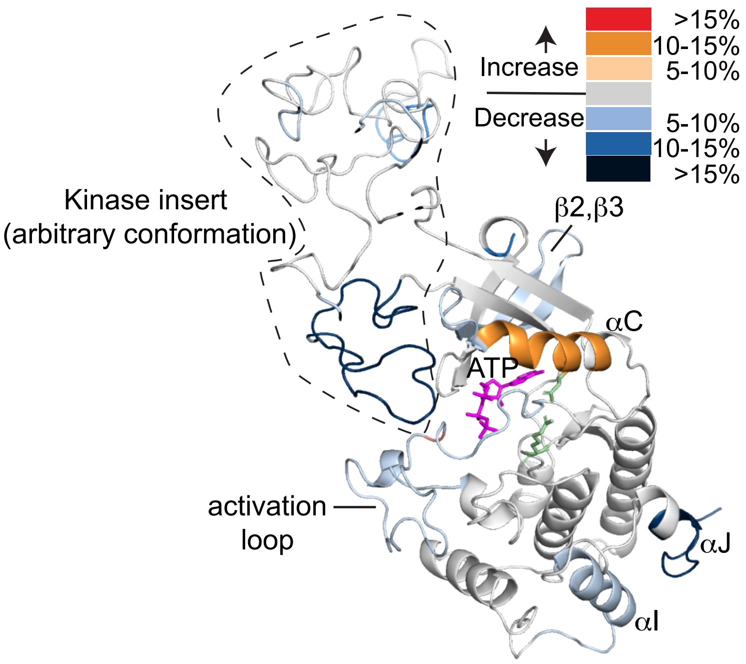

S5B Human GCN2 C-terminal domain colored by HDX differences

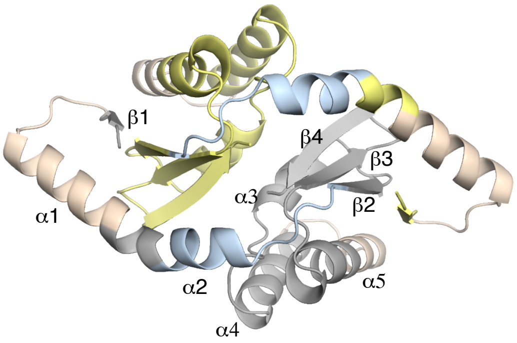

Fig. S5. Changes in HDX for GCN2 kinase and C-terminal domains

Changes in HDX for GCN2 resulting from P-stalk binding are shown mapped onto models of the structures of the human GCN2 kinase (**A**) and C-terminal domains (CTD) (**B**). The model for human GCN2 kinase domain was generated by SWISS-MODEL (6) based on the structure of yeast GCN2 domain (PDB ID 1ZYD) (7). The model for the kinase insert domain that is not part of the yeast kinase domain structure is arbitrary. The model for the human GCN2 CTD dimer is based on the structure of the murine CTD dimer (PDB ID 4TON) (8). To clarify the dimeric arrangement of the CTD, the base color for one protomer in the dimer is yellow.

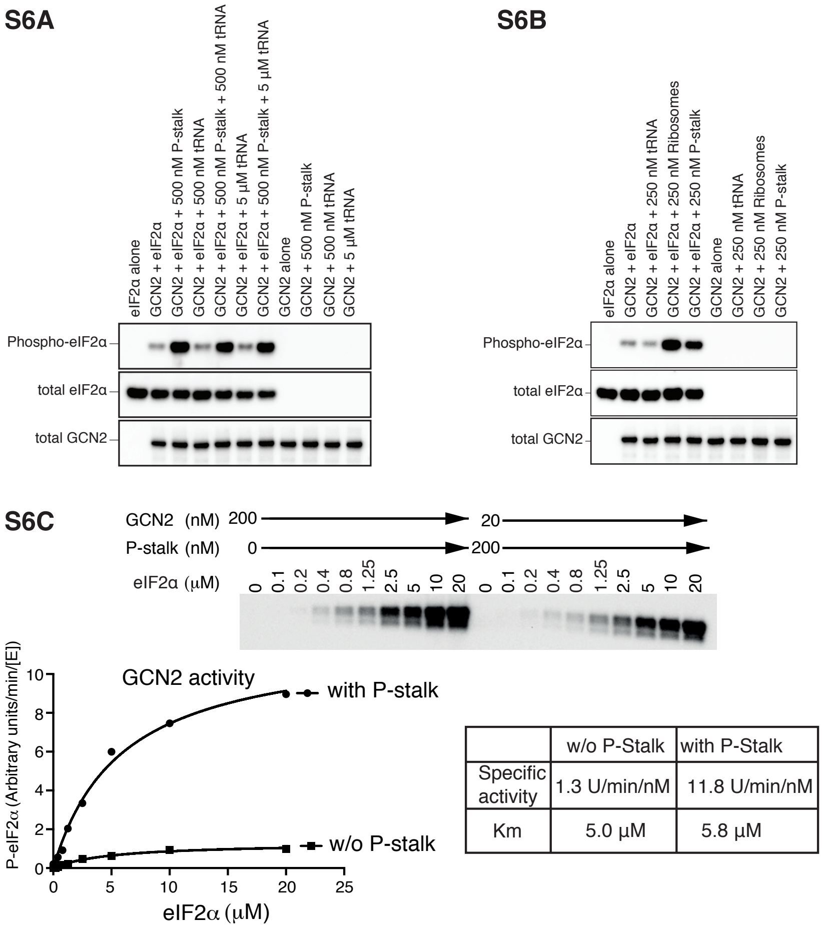

	w/o P-Stalk	with P-Stalk
Specific activity	1.3 U/min/nM	11.8 U/min/nM
Km	5.0 µM	5.8 µM

Fig. S6. Comparison of the effects of different regulators on GCN2 activation.

A. A comparison of the effect of the ribosomal P-stalk and deacylated tRNA alone, and in combination on GCN2-mediated eIF2α phosphorylation. The reactions were begun by the addition of ATP and then quenched after 5 minutes. The results were analysed by SDS-PAGE and Western blotting with antibodies against the GCN2 Strep-tag, P-eIF2α and total eIF2α.

B. The ability of different regulators to activate GCN2. GCN2-mediated eIF2α phosphorylation was assayed in the presence of either deacylated tRNA, purified ribosomes, or recombinant P-stalk (each at 250 nM), using the same assay as in panel A.

C. Basic kinase kinetic parameters for stimulated and unstimulated GCN2 with eIF2α substrate. Addition of the P-stalk increased the specific activity, but the Km remained largely unchanged.

Construct ID	Plasmid name	Construct boundaries	Description	Yield (mg/L cell culture)
A	Alp23	1-1649	Full length	1.5
B	Alp47	585-1024	KD	0.5
C	Alp42	1-1492	RWD−YKD−KD−HisRS -like	1
D	Alp40	192-1649	YKD−KD−HisRS-like−CTD	1
E	Alp44	192-1492	YKD−KD−HisRS-like	2
F	Alp58	260-1492	YKD−KD−HisRS-like	8
G	Alp46	192-539	YKD	1
H	Alp50	260-539	YKD	1
I	Alp43	192-1024	YKD−KD	5
J	Alp52	260-1024	YKD−KD	4
K	Alp59	585-1492	KD−HisRS-like	1
L	Alp60	585-1649	KD−HisRS-like−CTD	2
M	Alp57	1-1649 (F1143L R1144L)	m2 mutant	0.1
N	Alp36	1-1649 (D848N)	kinase dead mutant	2

Table S1. Details of the constructs from the GCN2 truncation library. A table giving the details of the plasmids for the GCN2 truncation library. The constructs' name corresponds to the construct names in the text. The construct boundaries, description and average yield per litre of cell culture are also given.

Additional data dataset S1 (separate file)

Dataset S1.: Summary of LC/MS/MS observations for peptides used in the HDX-MS analysis of GCN2 binding to ribosomes. For determination of HDX for ribosomal peptides, there were two reactions: one consisting of ribosomes alone at 0.5 µM, and a second consisting of ribosomes at 0.5 µM and GCN2 at 2.5 µM. Each ribosomal protein has a separate tab in the Excel file, and the overall statistics for the dataset are given on the first tab.

Additional data table S2 (separate file)

Dataset S2 Summary of LC/MS/MS observations for peptides used in the HDX-MS analysis of GCN2 binding to the P-stalk. For determination of HDX for P-stalk complex peptides, there were two reactions: one consisting of P-stalk complex alone at 5 µM, and one consisting of P-stalk complex at 5 µM and GCN2 at 15 µM. For determination of HDX for GCN2 peptides, there were also two reactions: one consisting of GCN2 alone at 5 µM, and one consisting of GCN2 at 5 µM and the P-stalk complex at 15 µM. Each protein has a separate tab in the Excel file, and the overall statistics for the dataset are given on the first tab.

1. Weissmann F, et al. (2016) biGBac enables rapid gene assembly for the expression of large multisubunit protein complexes. *Proc Natl Acad Sci* 113(19):E2564–9.

2. Trowitzsch S, Bieniossek C, Nie Y, Garzoni F, Berger I (2010) New baculovirus expression tools for recombinant protein complex production. *J Struct Biol* 172(1):45–54.

3. Zaborske JM, et al. (2009) Genome-wide analysis of tRNA charging and activation of the eIF2 kinase Gcn2p. *J Biol Chem* 284(37):25254–25267.

4. Sharma A, Mariappan M, Appathurai S, Hegde RS (2010) In Vitro Dissection of Protein Translocation into the Mammalian Endoplasmic Reticulum. *Protein Secretion: Methods and Protocols*, Protein Secretion: Methods and Protocols. ed Economou A (Humana Press, Totowa, NJ), pp 339–363.

5. Silva JC, et al. (2005) Quantitative proteomic analysis by accurate mass retention time pairs. *Anal Chem* 77(7):2187–2200.

6. Waterhouse A, et al. (2018) SWISS-MODEL: homology modelling of protein structures and complexes. *Nucleic Acids Res* 46(W1):W296–W303.

7. Padyana AK, Qiu H, Roll-Mecak A, Hinnebusch AG, Burley SK (2005) Structural basis for autoinhibition and mutational activation of eukaryotic initiation factor 2alpha protein kinase GCN2. *J Biol Chem* 280(32):29289–29299.

8. He H, et al. (2014) Crystal structures of GCN2 protein kinase C-terminal domains suggest regulatory differences in yeast and mammals. *Journal of Biological Chemistry* 289(21):15023–15034.
